# Ecology, threats and conservation status of *Carex buekii* (Cyperaceae) in Central Europe

**DOI:** 10.1038/s41598-019-47563-0

**Published:** 2019-08-01

**Authors:** Helena Więcław, Kateřina Šumberová, Beata Bosiacka, Richard Hrivnák, Zygmunt Dajdok, Attila Mesterházy, Chiara Minuzzo, Edoardo Martinetto, Jacob Koopman

**Affiliations:** 1 Institute of Marine and Environmental Sciences, University of Szczecin, Adama Mickiewicza 18, PL-70-383 Szczecin, Poland; 2 The Czech Academy of Sciences, Institute of Botany, Department of Vegetation Ecology, Lidická 25/27, CZ-602 00 Brno, Czech Republic; 3 Institute of Botany, Plant Science and Biodiversity Center, Slovak Academy of Sciences, Dúbravská cesta 9, SK-845 23 Bratislava, Slovakia; 4 Institute of Environmental Biology, University of Wrocław, Kanonia 6/8, PL-50-328 Wrocław, Poland; 5 Directorate of Hortobágy National Park, H-4024 Sumen utca 2, Debrecen, Hungary; 6 Regione Serramonte 10, I-10010 Andrate, Italy; 7 Earth Sciences Department, University of Turin, Via Valperga Caluso 35, I-10125 Turin, Italy; 8 ul. Kochanowskiego 27, PL-73-200 Choszczno, Poland

**Keywords:** Plant sciences, Ecology

## Abstract

*Carex buekii* is a tall sedge, forming large stands in wetlands, particularly in river floodplains across Central Europe and thus on many sites determining the typical appearance of riverine habitats. Our paper aims at increasing the knowledge on ecology of *C. buekii* and its role in the wetlands. Field data were collected in Poland, the Czech Republic, Slovakia, Hungary, and Italy. *Carex buekii* usually occurs in nutrient rich habitats, but is also capable of colonising relatively nutrient-poor ones; it grows on both acidic and alkaline soils (pH 3.3–7.4) with diverse concentrations of assimilable elements (Ca, Mg, P, K). One of the most important ecological characteristics of *C. buekii* is its relationship to the floodplains of watercourses. It seems to be dependent on, or at least very tolerant to regular disturbances by streaming, floods and transport of sediments. *Carex buekii* usually forms relatively uniform stands of its own association, *Caricetum buekii*. The species most frequently accompanying *C*. *buekii* are *Urtica dioica*, *Calystegia sepium*, *Galium aparine*, *Rubus caesius*, *Phalaris arundinacea*, and *Cirsium arvense*. The sedge also occurs in the understorey of forests with e.g. *Alnus glutinosa*, *Salix fragilis*, *Padus avium*, and *Quercus robur*. *Carex buekii* is able to colonise man-made or man-changed habitats such as railway embankments and roadsides or regulated river banks. Taking into account the IUCN Red List Criteria we propose to regard *C*. *buekii* as a least-concern (LC).

## Introduction

*Carex buekii* belongs to the section *Phacocystis*, one of the largest sections of the genus *Carex*, with about 90 species worldwide^1^, including about 16 species in Europe^2^. The section is taxonomically very difficult due to indistinct morphological differentiation between taxa and frequent hybridisation^3^. Consequently, *Carex buekii* is frequently confused with other members of the section *Phacocystis*, usually with *C*. *acuta*, *C*. *elata* and *C*. *randalpina*^4,5^. In spite of these facts, *Carex buekii* is relatively well distinguishable on account of its dark reddish-brown basal leaf sheaths which display a characteristic reticulate-fibrous structure, the shiny upper side of the leaves and its nerveless or indistinctly nerved utricles with very short beaks^2,6^.

The sedge occurs in central-eastern Europe, in the northern part of the Balkan Peninsula and in south-eastern Asia^7^. *Carex buekii* is included in the group of “river corridor plants”^8^. It usually grows on river banks along large and medium-sized river beds as well as on floodplains and valley slopes^9–11^. *Carex buekii* is also found in man-made habitats, such as ditch and canal banks, bridgeheads and river flood embankments, and roadsides^12,13^.

*Carex buekii* usually forms dense patches of plant communities classified in the phytosociological literature as *Caricetum buekii* Hejný et Kopecký in Kopecký et Hejný 1965 within the class *Phragmito-Magnocaricetea* Klika in Klika et Novák 1941^14^. This association has been recorded in Austria^15^, Croatia^16^, the Czech Republic^17,18^, Germany^19,20^, Hungary^10,21^, Poland^22^, and Slovakia^12,23^. The sedge has also been encountered as an accompanying species in wet meadow communities (*Calthion* Tüxen 1937, *Filipendulion* Segal 1966) as well as an understorey species in floodplain forests (*Alno-Ulmion* Br.-Bl. et R.Tx. 1943, *Salicion albae* R. Tx. 1955). For example, the association *Filipendulo ulmariae-Caricetum buekii* Háberová ex Balátová-Tuláčková in Rybníček *et al*. 1984 has been described from stream alluvia in the Slovenský kras Mts (southern part of Central Slovakia)^24,25^. Milenović and Ranđelović^26^ distinguished a forest association, *Carici buekii-Alnetum glutinosae*, which was invalidly described, as no type relevé was selected (see art. 5 of the International Code of Phytosociological Nomenclature (IPCN)^27^), from the upstream section of the River Sokobanjska Moravica (Serbia, the right-bank tributary of the South Morava, in the River Danube basin).

The ecology of *C*. *buekii* is rather poorly known and data on habitats with the occurrence of the species have been obtained from a few European countries only^12,28^. Therefore, it is difficult to evaluate its sensitivity to the recent global environmental changes, e.g. the climate change, land use change or overall eutrophication. On the other hand, it is likely that either retreat or spread of this species might have important implications for riverine wetland biodiversity, intensity of erosions, nutrient loadings and other processes.

Since the literature contains scant information on the ecology of the sedge, the present study was aimed at: (i) analysis of habitat requirements of *C*. *buekii* and its role in wetlands, (ii) exploring relationships between species composition in *C*. *buekii*-containing assemblages and habitat condition, (iii) identifying current threats and conservation needs of *C*. *buekii* in Central Europe.

## Material and Methods

### Field sampling

**Figure 1:**
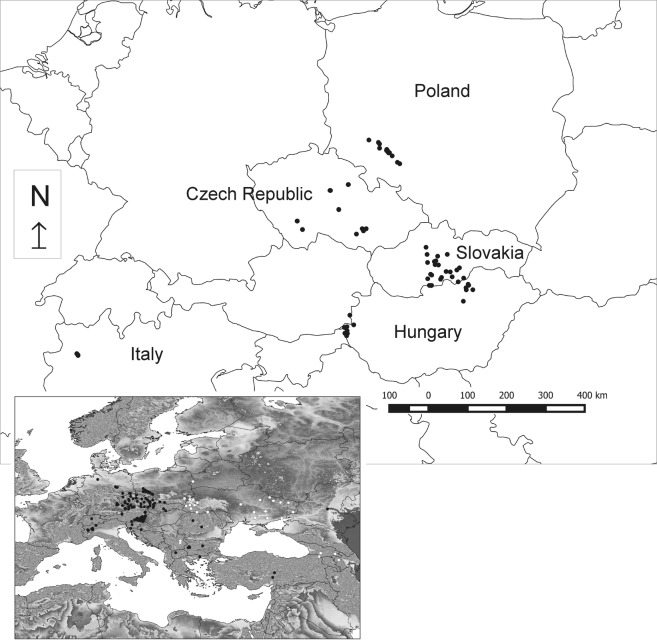
Locations of *Carex buekii* sampling sites and global distribution of *Carex buekii* (in the bottom left corner; according to Koopman *et al*.^7^; black circles – herbarium data, white circles – data from literature).

Field data were collected from May through July 2016 in Poland (20 plots), the Czech Republic (20 plots), Slovakia (21 plots), and Hungary (19 plots); in addition, the data set was supplemented by information from northern Italy (4 plots) (Fig. 1; Supplementary Tables S1 and S2). Only stands with a dominance of *Carex buekii* were recorded. The area of vegetation plots was usually in the range 30–100 m^2^. In order to obtain better insight into the ecology of *C*. *buekii* we decided to include in our research a few small plots from Poland (4 plots, 20–25 m^2^) and the Czech Republic (6 plots, 12–24 m^2^). The species cover in each plot was estimated using a nine-grade scale^29^. For each plot, three samples of soil were collected with Egner’s soil sampler from the plant root zone (0–25 cm). After mixing, they formed a single soil sample representing a given plot and intended for chemical analyses. The plant nomenclature follows Mirek *et al*.^30^. The nomenclature of the communities was not unified according to a single source and therefore at least the first use of each syntaxon is accompanied by the author’s name and year of the description. The names have been checked for validity according to IPCN^27^.

### Sources of climatic data and soil analyses

Climatic data for the studied regions have been taken from the national climate/environmental atlases or databases (Supplementary Table S1).

Soil samples were dried at room temperature and sieved to remove fractions larger than 1 mm. The following properties were determined: organic matter content (as loss on ignition at 550 °C), pH (potentiometrically in 1 M KCl), concentrations of assimilable forms of: phosphorous (P), potassium (K), magnesium (Mg), and calcium (Ca) using the American Society of Agronomy method, electrolytic conductivity of the saturated soil extract (ECe, conductometrically), total organic carbon (C), and nitrogen (N) (CHNS analyser, Costech Analytical Technologies Inc.)^31^. The last assay provided data for calculation of the ratio between C and N (C/N). A granulometric analysis was carried out according to Bouyoucos’s areometric method in the Casagrande and Prószyński modification^32^.

### Data analysis

The basic statistical metrics (arithmetic mean, minimum and maximum values) were determined for each of the studied soil properties, separately for data from Poland, the Czech Republic, Slovakia, Hungary, and Italy. The Shannon’s diversity index and the evenness were calculated for each relevé, using the MVSP package^33^. Relations between plot size and species number, evenness and Shannon’s diversity index were assessed on the basis of Spearman’s correlation coefficient. Statistical significance of differences between empirical distributions of the data analysed and the theoretical normal distribution was examined using the Shapiro–Wilk test. Since distributions of most data sets deviated from normal, the non-parametric Kruskal–Wallis test and Dunn’s multiple comparison test were used to examine whether differences between sites of *C*. *buekii* and between the diversity and evenness indices were significant. Calculations and graphs were prepared using the software package Statistica v. 12.0 for Windows^34^.

Plant species distribution patterns in relation to environmental variables were analysed by the canonical correspondence analysis (CCA), after the detrended correspondence analysis (DCA) had detected a unimodal structure of the species data (the gradient length represented by the first ordination axis higher than 3 SD). The environmental data were not transformed.

Tests of significance of the first and all canonical axes were performed for the statistical assessment of the relationship between the plant species composition and environmental variables (Monte Carlo test: 499 permutations under reduced model).

The Monte Carlo permutation test was further applied to test the statistical significance of environmental variables in explaining the plant species composition. For this purpose, stepwise “forward selection” of explanatory variables was used. The software package CANOCO v. 4.5 was used for the analyses^35^.

### Evaluation of threat level

The status of *C*. *buekii* was assessed using the IUCN Red List Criteria^36^, which are the world’s most widely accepted system for measuring extinction risk. The assessment has followed the Guidelines for Application of IUCN Red List Criteria at Regional Level^37^. The criteria for evaluating the level of endangerment of plant communities (i.e. current status, past trend and prognosis) has followed Berg *et al*.^38^.

## Results

### Habitat conditions

**Figure 2:**
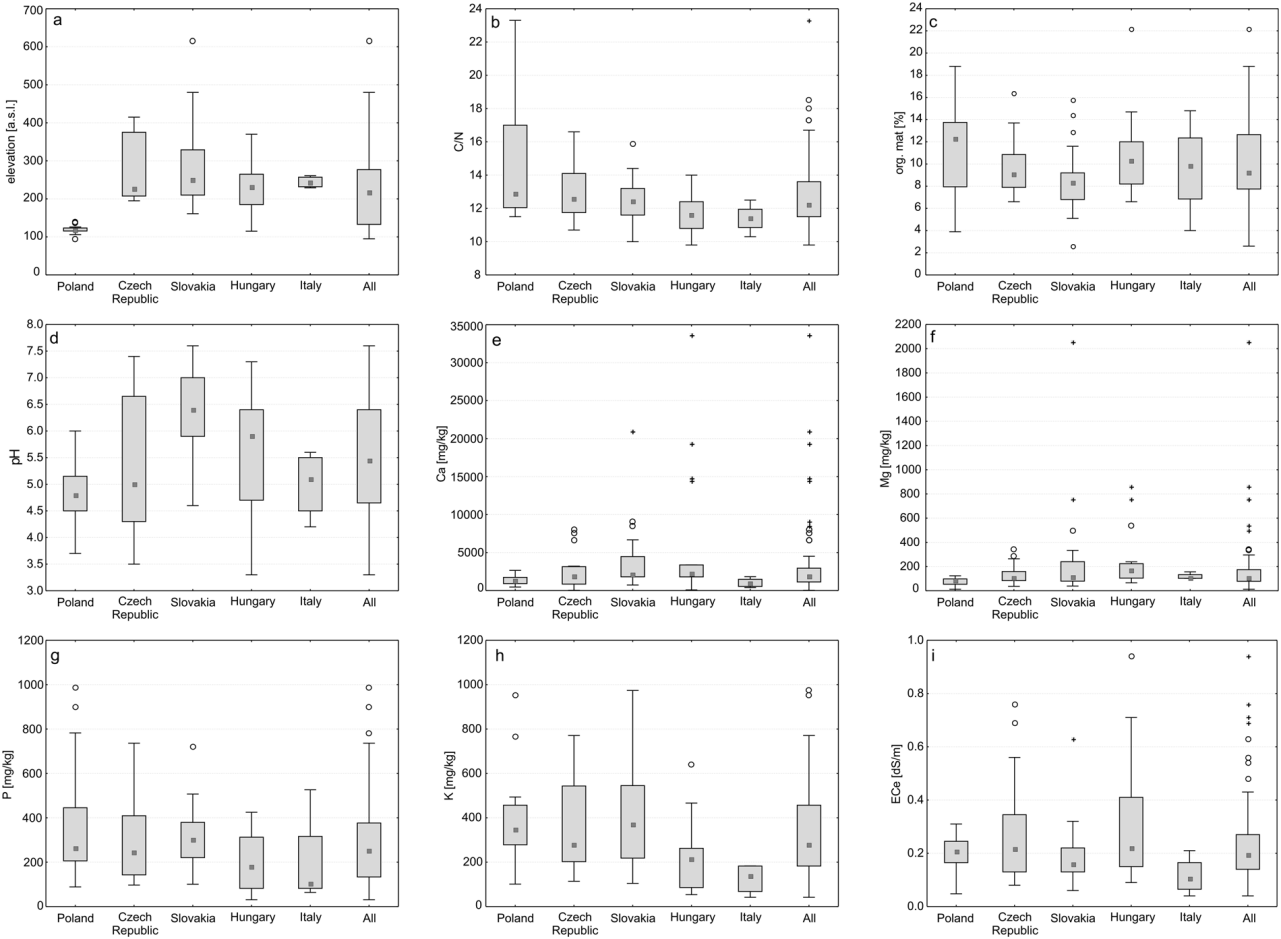
Ranges of environmental variables at *C*. *buekii* sites. Large boxes indicate 25–75% of the interquartile ranges; small boxes - medians; white circles - outliers; asterisks - extreme values. (**a**) Elevation; (**b**) Ratio between organic carbon and nitrogen concentration; (**c**) Organic matter content; (**d**) Soil pH; (**e**) Calcium concentration; (**f**) Magnesium concentration; (**g**) Phosphorus concentration; (**h**) Potassium concentration; (**i**) Electrolytic conductivity of the saturated soil extract.

*Carex buekii* is a lowland species; most of the localities in this study were situated at elevations of 95–480 (−616) m (Fig. 2a; Supplementary Table S1).

The mean annual temperature at *C*. *buekii* sites usually ranged from 7 °C to 12 °C and the annual sum of precipitation from 500 mm to 800 mm (Supplementary Table S1).

The C/N mean for all samples was 12.8 and ranged from 9.8 to 18.5 (−23.3) (Table 1; Fig. 2b). Most soil samples can be described as mineral-humic, except for some samples from Poland, being organic-mineral.

**Table 1:** Soil properties at *Carex buekii* sites. P – Poland, CR - Czech Republic, S – Slovakia, H – Hungary, I – Italy; C/N – ratio between organic carbon and nitrogen concentration; org. mat. – organic matter content; pH – soil pH; Ca – calcium concentration; Mg – magnesium concentration; P – phosphorus concentration; K – potassium concentration; ECe – electrolytic conductivity of the saturated soil extract; $\bar{{\rm{x}}}$ – mean; SD – standard deviation; range – minimum and maximum values.

Country	Soil properties							
	C/N		org. mat [%]		pH		Ca [mg/kg]	
	range	$\bar{{\rm{x}}}$ ± SD	range	$\bar{{\rm{x}}}$ ± SD	range	$\bar{{\rm{x}}}$ ± SD	range	$\bar{{\rm{x}}}$ ± SD
P	11.5–23.3	14.4 ± 3.185	3.9–18.8	11.3 ± 3.950	3.7–6.0	4.9 ± 0.545	462.0–2654.7	1395.0 ± 656.518
CR	10.7–16.6	12.9 ± 1.484	6.6–16.4	9.7 ± 2.531	3.5–7.4	5.3 ± 1.308	34.2–8079.3	2511.9 ± 2323.291
S	10.0–15.9	12.5 ± 1.453	2.6–15.8	8.6 ± 3.125	4.6–7.6	6.4 ± 0.886	731.0–20943.1	4004.7 ± 4514.962
H	9.8–14.0	11.6 ± 1.079	6.6–22.2	10.8 ± 3.688	3.3–7.3	5.6 ± 1.239	86.5–33633.3	5836.4 ± 8666.034
I	10.3–12.5	11.4 ± 0.898	4.0–14.8	9.6 ± 4.416	4.2–5.6	5.0 ± 0.632	396.7–1823.6	1015.2 ± 620.935
	**Mg [mg/kg]**		**P [mg/kg]**		**K [mg/kg]**		**ECe [dS/m]**	
	**range**	$\bar{{\rm{x}}}$** ± SD**	**range**	$\bar{{\rm{x}}}$** ± SD**	**range**	$\bar{{\rm{x}}}$** ± SD**	**range**	$\bar{{\rm{x}}}$** ± SD**
P	12.3–123.4	78.2 ± 28.653	88.5–985.4	362.2 ± 257.432	101.2–950.8	385.3 ± 194.776	0.05–0.31	0.20 ± 0.062
CR	36.5–342.0	133.3 ± 84.227	96.8–736.4	313.4 ± 202.037	114.0–770.9	359.6 ± 212.045	0.08–0.76	0.28 ± 0.201
S	38.8–2056.0	272.8 ± 443.076	100.7–721.6	298.5 ± 149.019	104.4–974.0	401.9 ± 232.892	0.06–0.64	0.19 ± 0.119
H	65.4–861.1	233.4 ± 228.223	31.0–425.1	193.2 ± 120.153	53.8–638.4	210.1 ± 148.549	0.09–0.94	0.30 ± 0.220
I	102.6–156.1	117.9 ± 25.663	63.7–526.3	199.3 ± 218.837	41.8–183.1	125.3 ± 69.622	0.04–0.21	0.12 ± 0.071

The mean organic matter content (about 10.0%) was similar for most of the samples. Outlying values were determined for the Slovak and Hungarian sites (2.6 and 22.2%, respectively) (Table 1; Fig. 2c).

The soil pH was found to span a wide range, from strongly acidic (16%) to alkaline (21%). Most soil samples (63%), however, were acidic or slightly acidic (pH 4.5–6.5, mean 5.5). The Slovak samples were distinct in their relatively high pH values, with a maximum of 7.6 (Table 1; Fig. 2d).

The mean concentrations of assimilable forms of elements of *C*. *buekii* sites were: 3299.9 mg/kg (range 34.2–33633.3) for Ca, 176.9 mg/kg (range 12.3–2056.0) for Mg, 288.7 mg/kg (range 31.0–985.0) for P and 331.3 mg/kg (range 41.8–974.0) for K (Fig. 2e–h; Table 1). In terms of concentrations of assimilable elements in the soil samples from countries examined, (i) the highest mean concentrations of Ca and Mg were detected in the Slovak and Hungarian samples (some of which showed outlying values); (ii) the mean P concentration was high in the samples collected in Poland compared to the other countries (there were two outliers with the highest P concentrations among the samples); (iii) the highest K concentration was recorded in the Slovak samples, the lowest being found in samples from Hungary and Italy (Table 1; Fig. 2e–h).

The soil conductivity expressed as conductivity of the soil saturated extract was very low; all the samples showed a values below 1 dS·m^−1^ (Table 1; Fig. 2i).

**Figure 3:**
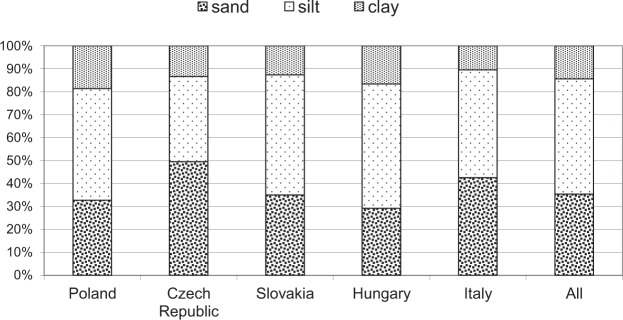
Comparison of soil fractions at *Carex buekii* sites.

The soil of all habitats examined consisted mainly of muddy and clayey silts (50%), the remainder fractions representing sand and different clays (Fig. 3). The highest content of clay was found in the Polish and Hungarian samples, while the highest content of silts in the Slovak and Hungarian samples. Significant differences in clay content were revealed between samples from Poland and the Czech Republic as well as between samples from Poland and Slovakia (Supplementary Table S3). Significant differences in silts content were found between samples from Hungary and the Czech Republic as well as between samples from Slovakia and the Czech Republic.

The results of the Kruskal–Wallis test and *post hoc* Dunn’s multiple comparisons test indicated that the *C*. *buekii* stands in the investigated countries vary significantly in eleven environmental variables (elevation, precipitation, temperature, soil pH, soil fractions, C/N ratio, and the concentrations of K, Ca and Mg) (Supplementary Table S3). The highest number of statistically significant differences was found between Polish and Slovak samples as well as between those collected in Poland and Hungary.

### Diversity of plant communities with *Carex buekii*

*Carex buekii* is as a dominant species within the *Caricetum buekii* association. The vegetation samples examined were found to contain a total of 178 species; 83 species occurred in less than 3 plots (47% of the species turned sporadic). The species most frequently accompanying *C*. *buekii* were *Urtica dioica* (61% of relevés), *Calystegia sepium* (45%), *Galium aparine* (40%), *Rubus caesius* (36%), *Phalaris arundinacea* (35%), and *Cirsium arvense* (32%) (Supplementary Table S2). In addition, more than 50% of the Polish, Slovak, and Hungarian vegetation samples contained *Vicia cracca*, *Galium rivale* and *Solidago gigantea*, respectively. The phytocoenoses with *C*. *buekii* were relatively species poor; overall, an average of 10 species per phytosociological relevé was recorded (Supplementary Table S4). The value of the Shannon’s diversity index ranged from 0, in single-species patches (relevés 22, 23 and 36) up to 4.25 (mean 2.82) in patches composed of more than 20 species (e.g. relevé 71) (Supplementary Tables S1 and S4).

The Hungarian, Polish and Italian vegetation samples showed a higher species richness (mean number of species 14, 12 and 11, respectively), a higher biodiversity index (equal or higher than 3.20) and a more even distribution of species between assemblages (evenness equal or higher than 0.90), compared to the plots sampled in the Czech Republic and Slovakia (Supplementary Table S4). The Polish and Czech relevés differed significantly in terms of species richness, diversity and evenness. Low correlations between plot size and species number, evenness and Shannon’s diversity index were detected (Spearman correlation coefficients = 0.4006, 0.2172 and 0.3917, respectively).

Most of the phytocoenoses were growing in open-terrain, rush-vegetated areas. Moist forests in which *C*. *buekii* was the understorey dominant, yielded 14 relevés (13 in tree stands with *Alnus glutinosa*, *Padus avium*, *Salix fragilis*, and *Salix alba* in Hungary; one relevé in a tree stand with *Quercus robur* in Poland). In addition, 3 vegetation samples in Italy showed a single occurrence of the tree species mentioned above. Some other relevés, e.g. from the rivers Svratka, Svitava and Orlice in the Czech Republic, have been situated on the forest margins, thus being influenced by shading (Supplementary Table S1) and by the transition of forest species such as *Aegopodium podagraria*, *Impatiens parviflora* and *Stellaria nemorum*.

### Distribution of plots and species along environmental gradients

All the variables included in CCA accounted for 18.23% of the total variance in the species data (Table 2). All the canonical axes were significant as tested by the unrestricted Monte Carlo permutation test (the first axis: *p* = 0.021; all axes: *p* = 0.005).

**Table 2:** CCA summary for samples collected in Poland, Czech Republic, Slovakia, Hungary, and Italy.

Axes	I	II	II	IV
Eigenvalues	0.293	0.187	0.149	0.137
Species-environment correlations	0.895	0.842	0.789	0.774
Cumulative percentage variance of species data	4.3	7.0	9.1	11.1
Cumulative percentage variance of species-environment relation	23.3	38.1	50.0	60.90
Sum of all eigenvalues/ Total inertia	6.880			
Sum of all canonical eigenvalues	1.258			
Percentage of explained species data variance	18.23			

The results of the forward selection of variables revealed seven variables (precipitation, pH, organic matter content, elevation, temperature, K and P) to be statistically significant, and accounting for 13.4% of the total variance in the species composition (Table 3).

**Table 3:** Forward selection results with the test of variable significance for samples collected in Poland, Czech Republic, Slovakia, Hungary, and Italy. *statistically significant variables (p ≤ 0.05). For abbreviations of soil properties, see Table 1.

Variables	LambdaA	Explained data variance [%]	F-ratio	p-value
precipitation*	0.25	3.6	3.10	0.002
pH*	0.14	2.0	1.69	0.004
org.mat*	0.12	1.7	1.55	0.012
temperature*	0.12	1.7	1.49	0.008
elevation*	0.11	1.6	1.42	0.014
K*	0.10	1.4	1.35	0.045
P*	0.10	1.4	1.32	0.048
EC_e_	0.09	1.3	1.29	0.158
Ca	0.09	1.3	1.26	0.248
C/N	0.09	1.3	1.21	0.198
Mg	0.04	0.6	0.53	0.928

**Figure 4:**
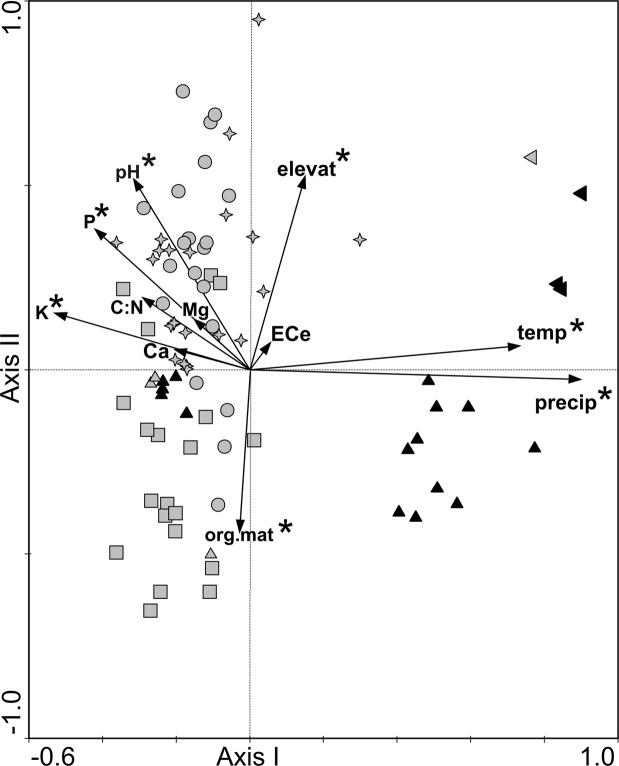
Ordination diagram of vegetation samples and environmental variables along the first two CCA axes. Poland – boxes, the Czech Republic – circles, Slovakia – asterisks, Hungary – up-triangles, Italy – left-triangles; grey symbols – open-terrain samples, black symbols – forest and shrub samples; elevat – elevation; temp – mean annual temperature; precip – annual sum of precipitation; *statistically significant variables. For abbreviations of soil properties, see Table 1.

The results of CCA showed different position of samples in ordination space depending on the studied region (Fig. 4). The lower right quarter of the diagram contains Hungarian forest samples formed by *Alnus glutinosa*, *Salix alba* and *Salix fragilis*, related to relatively high values of precipitation and temperature, moderate values of organic matter content and elevation, and relatively low and moderate values of pH, and K and P concentrations. This part of the diagram additionally features one Polish forest sample formed by *Quercus robur* in a tree layer. The upper right quarter of the diagram contains samples from Italy, including three phytocoenoses with scattered occurrence of trees, *Quercus robur, Alnus glutinosa* and *Salix alba*, related to relatively high values of precipitation, temperature and elevation, moderate values of pH and P concentrations, and relatively low values of organic matter content and K concentrations. Five forest samples placed in the lower left quarter of the diagram are obtained from Hungarian forests with occurrence of *Padus avium* in the tree layer, related to relatively low values of precipitation and temperature, moderate values of organic matter content and pH, and relatively high values of K and P concentrations. Most of the phytocoenoses with *C. buekii* growing in open-terrain, rush-vegetated areas, are located in the left part of the ordination space. The upper left quarter of the diagram contains non-forest samples mainly from the Czech Republic and Slovakia, related to relatively low values of precipitation and temperature, low and moderate values of organic matter content, relatively high and moderate values of pH and elevation, and relatively high values of P and K concentrations. Most samples collected in Poland are located in the lower left quarter of the diagram, including non-forest phytocoenoses related to relatively low values of precipitation and temperature, high and moderate values of organic matter content, P and K concentration, and relatively low and moderate values of pH and elevation (Fig. 4).

**Figure 5:**
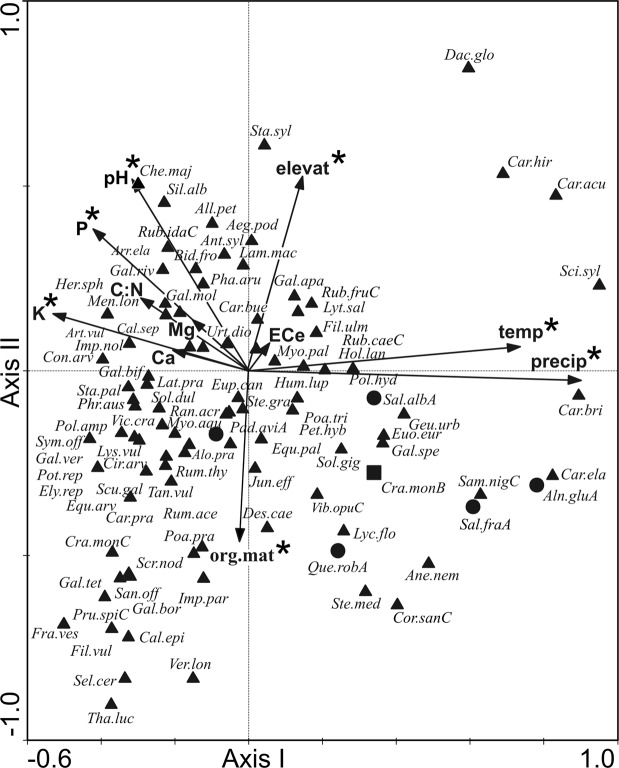
Ordination diagram of species and environmental variables along the first two CCA axes. Herb layer – black triangles; shrub layer – black squares, tree layer – black circles; precip – annual sum of precipitation; elevat – elevation; temp – mean annual temperature; *statistically significant variables. For abbreviations of soil properties, see Table 1. Abbreviations (96 species, without 83 sporadic species): *Aeg.pod - Aegopodium podagraria, All.pet - Alliaria petiolata, Aln.gluA - Alnus glutinosa* (tree layer)*, Alo.pra - Alopecurus pratensis, Ane.nem - Anemone nemorosa, Ant.syl - Anthriscus sylvestris, Arr.ela - Arrhenatherum elatius, Art.vul - Artemisia vulgaris, Bid.fro - Bidens frondosa, Cal.epi - Calamagrostis epigeios, Cal.sep - Calystegia sepium, Car.acu - Carex acutiformis, Car.bri - Carex brizoides, Car.bue - Carex buekii, Car.hir - Carex hirta, Car.ela - Carex elata, Car.pra - Carex praecox, Che.maj - Chelidonium majus, Cir.arv - Cirsium arvense, Con.arv - Convolvulus arvensis, Cor.sanC - Cornus sanguinea* (herb layer)*, Cra.monB - Crataegus monogyna* (shrub layer), *Cra.monC - Crataegus monogyna* (herb layer), *Dac.glo - Dactylis glomerata, Des.cae - Deschampsia caespitosa, Equ.arv - Equisetum arvense, Equ.pal - Equisteum palustre, Euo.eur - Euonymus europaeus, Eup.can - Eupatorium cannabinum, Fil.ulm - Filipendula ulmaria, Fil.vul - Filipendula vulgaris, Fra.ves - Fragaria vesca, Gal.bif - Galeopsis bifida, Gal.spe - Galeopsis speciosa, Gal.tet - Galeopsis tetrahit, Gal.apa - Galium aparine, Gal.bor - Galium boreale, Gal.mol - Galium mollugo, Gal.riv - Galium rivale, Gal.ver - Galium verum, Geu.urb - Geum urbanum, Her.sph - Heracleum sphondylium, Hol.lan - Holcus lanatus, Hum.lup - Humulus lupulus, Hyp.per - Hypericum perforatum, Imp.nol - Impatiens noli-tangere, Imp.par - Impatiens parviflora, Jun.effJuncus - effusus, Lam.mac - Lamium maculatum, Lat.pra - Lathyrus pratensis, Lyc.flo - Lychnis flos-cuculi, Lys.vul - Lysimachia vulgaris, Lyt.sal - Lythrum salicaria, Men.lon - Mentha longifolia, Myo.pal - Myosotis palustris, Myo.aqu - Myososton aquaticum, Pad.aviA - Padus avium* (tree layer), *Pet.hyb - Petasites hybridus, Pha.aru - Phalaris arundinacea, Phr.aus - Phragmites australis, Poa.pra - Poa pratensis, Poa.tri - Poa trivialis, Pol.amp - Polygonum amphibium* f*. terrestre, Pol.hyd - Polygonum hydropiper, Pot.rep - Potentilla reptans, Pru.spiC - Prunus spinosa* (herb layer), *Que.robA - Quercus robur* (tree layer), *Ran.acr - Ranunculus acris, Rub.caeC - Rubus caesius* (herb layer)*, Rub.ida -Rubus idaeus, Rub.fruC - Rubus fruticosus* agg. (herb layer), *Rum.ace - Rumex acetosa, Rum.thy - Rumex thyrsiflorus, Sal.albA - Salix alba* (tree layer), *Sal*.*fraA* –*Salix fragilis* (tree layer), *Sam.nigC - Sambucus nigra* (herb layer), *San.off - Sanguisorba officinalis, Sci.syl - Scirpus sylvaticus, Scr.nod - Scrophularia nodosa, Scu.gal - Scutellaria galericulata, Sel.cer - Selinum carvifolia, Sil.alb - Silene alba, Sol.dul - Solanum dulcamara, Sol.gig - Solidago gigantea, Sta.pal - Stachys palustris, Sta.syl - Stachys sylvatica, Ste.gra - Stellaria graminea, Ste.med - Stellaria media, Sym.off - Symphytum officinale, Tan.vul - Tanacetum vulgare, Tha.luc - Thalictrum lucidum, Urt.dio - Urtica dioica, Ver.lon - Veronica longifolia, Vib.opu - Viburnum opulusC* (herb layer), *Vic.cra - Vicia cracca*.

As shown by the ordination diagram of species and environmental variables, the maximum abundance of *C*. *buekii*, together with a group of most frequently accompanying species located in the central part of the diagram, is related to moderate values of all statistically significant variables (Fig. 5).

### Threat level of *Carex buekii*

The current evaluation of the species situation concerning Central Europe with the use of IUCN guidelines at regional level^37^ indicates that the category LC (Least Concern) is suitable for *C*. *buekii*. This sedge does not meet any of the A-E criteria defined in relation to the categories CR (critically endangered), EN (endangered) and VU (vulnerable). Although, *C*. *buekii* is classified as a river corridor plant and its habitats are severely fragmented, the extent of occurrence (EOO) and/or area of occupancy (AOO) are larger than the threshold given for the NT category^36^. Moreover, this sedge is able to colonise man-made or man-changed habitats such as railway embankments and roadsides or regulated river banks. These facts prove the adaptation abilities of this species.

The threat category of vegetation predominated by *C*. *buekii* was determined as LC (Least Concern). This category includes rare or infrequent plant communities, currently not threatened by human activities nor declining. The current status, i.e. the present number, distribution and size of the stands in the studied area, was estimated as infrequent (total area of stands moderately large and occurrence in 11–33% of the geographically defined units). The trend in the past, as constant (more or less constant situation or only minor local loss, ±10% range) and the prognosis, i.e. the properties of existing or foreseeable, directly or indirectly, effects the survival of a plant community type, as low negative direct or indirect impacts.

## Discussion

### Ecology of *Carex buekii*

According to Ellenberg *et al*.^39^, *C*. *buekii* requires sunny (Ellenberg indicator value, EIV for light = 8), moist (EIV = 8), nutrient rich (EIV = 6), and alkaline (EIV = 8) sites. Studies conducted by Heerde *et al*.^28^ revealed a need to verify the indicators shown above. The authors demonstrated that, in Germany, *C*. *buekii* can grow in habitats with a varying degree of insolation (the sedge occurred also at shaded sites), showing a wide range of humidity (even at considerably drier habitats) and diverse pH values. In the countries we obtained our data from, the sedge occurs both at open and sunny sites and in moderately shaded forest localities. *Carex buekii* is also highly tolerant to habitat moisture variation. For example, it grew, in a good condition, on inundated meadows as well as overdried soil of dykes and embankments^13^. The tolerance to variable moisture is most probably related to the sedge’s root length, as the roots can grow to as much as 3 m down in the soil^14^. The sedge usually occurred in nutrient rich habitats, but was also capable of colonising relatively nutrient-poor ones. Moreover, it seems that the occurrence of *C*. *buekii* is not limited by the soil pH. At the sites we investigated, the species grew on both acidic and alkaline soils; only in Slovakia the sedge was more frequently encountered on higher-pH soils^12^, however, this may be related to a generally rare occurrence of acidic soils in this country. Similarly, diverse concentrations of assimilable elements (Ca, Mg, P, K) in the soil did not limit the species’ occurrence. We observed diverse soil conditions both at open-terrain rush-covered *C*. *buekii* habitats and in forested ones. Among the types of soil which are probably avoided by *C. buekii*, are saline soils, representing extreme conditions for lots of plant species. All the samples have shown very low conductivity of the saturated soil extract which is typical of soils with low concentrations of soluble salts^40^. On the other hand, this sedge avoids peaty substrates, probably due to an extremely low amount of accessible nutrients. These relationships are also obvious from the distribution of *C. buekii* in all the countries; it grows neither in the regions with frequent occurrence of salt marshes nor in peatland areas^41^. More detailed ecological studies involving a larger data set from the entire distribution range of this sedge are doubtlessly required in the future. With data from these regions it will be possible to comprehensively define the ecological range of *C*. *buekii* on a global scale.

One of the most important ecological characteristics of *C. buekii* is its very strong relationship to the floodplains of watercourses, particularly of large rivers^8^. It seems to be dependent on, or at least very tolerant to regular disturbances by streaming, floods and transport of sediments^42^. Although it forms large stands, also on the sites relatively far away from active river beds and without regular floods, it is not clear if these occurrences may be stable from the long-term perspective (i.e. dozens of years). The stands on man-made (e.g. dykes) as well as natural and near-nature habitats subjected to recent land use and environmental changes (e.g. abandoned meadows, terrestralised floodplain pools and river arms) have not occurred in the landscape for sufficiently long time to enable any reliable conclusions. On the one hand, a lot of the populations have been repeatedly mapped on the same sites during the last 10–20 years. On the other hand, it cannot be excluded that very long sedimentation of a thick litter layer produced by *C. buekii* might finally lead to the lowering of vitality of its stands, similarly as in many other reed bed and tall sedge species^43^.

Our study sites are situated on the climatic gradient from south-western Poland (with the northernmost locality in the River Kaczawa valley at Kwiatkowice) to north-western Italy (with the southernmost locality in the River Malone valley at Front) well representing the climatic conditions in which *C. buekii* is able to grow^44^. Although the climate certainly has important implications for the development of any vegetation type, it seems to be relatively less important in some types of wetlands, particularly in river floodplains that usually exhibit a specific local climate compared to the surrounding landscape outside the floodplains. In this study, we have used the data available on macro-climatic conditions of the regions where the relevés were recorded. A better insight into the local climatic conditions directly on the study sites could be obtained by continual measurements using temperature sensors. Additionally, floodplains contribute not only to the buffering of climate extremes but also their soils usually originate in mixing soil particles from the broad surroundings. Therefore, it may be supposed that interactions between climate and soil properties (e.g. the amount of nutrients, calcium and soil pH), frequently modifying species’ distribution and habitat ecology on a large scale, are not very important in floodplains. Most common among the temperate European species and communities is the preference of nutrient and/or calcium-rich habitats in the regions with relatively cold and humid climate and either avoidance of these conditions, or widening of the ecological niche towards nutrient and calcium poorer habitats in relatively warm and dry climates^18,45^. Our data do not indicate any important relationship in this sense. Therefore, we suggest that habitat type, surrounding vegetation, management and partly also soil properties are the main forces shaping the overall appearance, dynamics and species composition of the vegetation predominated by *C. buekii*. Climatic factors, on the other hand, form the limits for the occurrence or non-occurrence of the species and its stands.

### Vegetation characteristic of *Carex buekii*

Despite differences in Shannon’s diversity and evenness, the floristic composition in stands with *C*. *buekii* dominance is generally similar in all the studied countries. In Europe, the species listed as those accompanying *C*. *buekii* most often include *Calystegia sepium*, *Urtica dioica*, *Phalaris arundinacea*, *Galium aparine*, *Cirsium arvense*, *Filipendula ulmaria*, *Rubus caesius*, and *Symphytum officinale*^16,20,21^. Thus, it seems that the vegetation dominated by *C*. *buekii* is relatively uniform within its distribution range which we confirmed also in this study. This result is not surprising because *C*. *buekii* is very strongly dominant, not allowing co-occurrence of many other species. Although the cover of herb layer usually does not reach 100%, a very dense root system and dense and thick litter layer form conditions unsuitable for the establishment of other species and thus monocoenoses of *C. buekii* are not rare. Whenever other species occur, these are usually common wetland, grassland and nitrophilous species, spreading from the surrounding vegetation. Most of the accompanying species are perennials with dispersal by vegetative propagules. Annual plant species are much less common, as the thick litter layer prevents seed germination and seedling recruitment^46^. Therefore, annuals such as *Bidens frondosa*, *Myosoton aquaticum* or *Galeopsis* spp. usually occur only in the stands where the continual litter layer is somewhat disturbed, e.g. by water or on resting sites of game. *Galium aparine* is the only frequent annual because, according to our experiences, it is able to germinate even on a thick layer of undecomposed biomass.

Despite the relatively low species diversity of its stands and unstable overall species composition (i.e. most of the species are treated just as accompanying ones, without any syntaxonomical value), *Caricetum buekii* has been placed in different higher syntaxa^16,21,47^. Kopecký and Hejný^14^ described the association *Caricetum buekii* and assigned it to the alliance *Phalaridion arundinaceae* Kopecký 1961 within the order *Nasturio-Glycerietalia* Pignatti 1953 and the class *Phragmito-Magnocaricetea* Klika in Klika et Novák 1941. However, the alliance *Phalaridion arundinacae* has not been accepted by many authors due to its poor floristic differenciation. Philippi^48^ placed *Caricetum buekii* in the alliance *Magnocaricion* Koch 1926, the order *Phragmitetalia* Koch 1926 and the class *Phragmito-Magnocaricetea*. Further, Ellmauer and Mucina^15^ assigned this plant community to the alliance *Calthion* Tüxen 1937, the order *Molinietalia* Koch 1926 and the class *Molinio-Arrhenatheretea* Tüxen 1937. Kopecký and Hejný^14^ recorded the presence of *C*. *buekii* under the canopy of trees and shrubs representing the genera *Salix* and *Alnus*. Similarly, Alegro and Markovič^9^ listed *C*. *buekii* as an understorey component of floodplain forests, but did not provide any clear indication of the syntaxonomic affiliation. Further, Milenović and Ranđelović^26^ identified a forest association *Carici buekii*-*Alnetum glutinosae* (invalid description according to IPCN, art. 5) and placed it in the class *Alnetea glutinosae* Br.-Bl. et Tüxen ex Westhoff *et al*. 1946. Also our own research shows *C*. *buekii* to be capable of growing in the understorey of floodplain forests formed by *Alnus glutinosa, Salix fragilis*, *Quercus robur*, *Padus avium*, and *Salix alba*. In summary, it should be emphasised that *C*. *buekii* is most often mentioned as a dominant species within the *Caricetum buekii* association, which, according to a majority of literature sources, is included in the *Phragmito-Magnocaricetea* class.

### Threats, conservation recommendations and ecosystem functions

*Carex buekii*’s capability of colonising anthropogenically altered sites such as dykes, banks of ditches and roadsides, may indicate the species’ tolerance to anthropogenic pressure. Direct destruction of phytocoenoses and habitats, as a result of changes in land use (disturbance of river valleys, earthworks associated with river regulation, embankment uplift, floodplain use as arable land, lowering of the groundwater level, etc.) seems to be the major threat for the sedge as well as for other species from the group of “river corridor plants”, e.g. *Allium schoenoprasum* (LC category according to IUCN Red List), *Alisma lanceolatum* (LC), *Cyperus michelianus* (LC), *Mentha pulegium* (LC), *Limosella aquatica* (LC), *Lindernia procumbens* (LC), *Salvinia natans* (LC), *Veronica catenata* (LC)^8,42^. In addition, construction of roads and associated infrastructure leads to habitat fragmentation and overdrying. Wet meadows in river valleys with a mass occurrence of *C*. *buekii* are generally subjected to overgrowth of shrubs and woody species. Although *C. buekii* is also found in forests, it occurs there in plots with scattered trees rather than with a compact tree canopy. Alien species, including those regarded as invasive, e.g. *Solidago gigantea*, often grow in communities predominated by *C*. *buekii*, shown by our data from Hungary, Poland and Slovakia. Most likely, however, anthropophytes do not pose a threat to *C*. *buekii* as these rather competitive species are mainly unable to compete with this perennial, tuft-forming sedge with its long and thick rhizomes.

Conservation of *C*. *buekii*-supporting communities requires elimination of activities which directly contribute to destruction of the species and its habitat. Although the stands of *C*. *buekii* do not seem to be very important for preservation of other plant species, its overall ecosystem function, e.g. its relationships to various groups of animals, fungi and micro-organisms, is still poorly known. The scarce studies suggest that *C. buekii* may serve as an important host plant for some groups of insects^49^. It should be emphasised that destructive anthropogenic activities represent a highly negative pressure also on the whole range of other species and plant communities related to floodplains. Some of them, for instance floodplain wet meadows, are very valuable from the point of view of wetland biodiversity conservation^50–52^ and they are even more threatened than *Caricetum buekii*. In contrast, they suffer from the spread of *Caricetum buekii* in floodplains after abandonment of mowing^16,18^. Thus, the optimal management of floodplain habitats should lead to a mosaic of various plant communities, offering suitable conditions for a high number of plant and animal species. For instance, floodplains with various non-forest vegetation types are known as biodiversity hot spots for *Odonata*^53^. Functions of typical floodplain communities in the landscape are indispensable; they do not only maintain unique organisms and a high level of biodiversity, but they also contribute to the protection against erosion and floods of large areas^54,55^.

### Conservation status

Schnittler and Günther^56^, who analysed national Red Lists and distribution maps in European countries, concluded that *C*. *buekii* is a species threatened in Europe, and placed it in the category vulnerable (VU). However, this sedge was not taken into account on the red list of vascular plants in Europe, published in 2011^57^.

In Poland, *C*. *buekii* has been placed both in the Red Book of Plants^13^ and on the Red List^58^ as a near-threatened (NT) species, although earlier Kopeć and Michalska-Hejduk^59^ suggested category CR (critically endangered). Most records of this sedge are from the south-western part of the country, from the Odra river valley in the vicinity of Wrocław^13,22^. *Carex buekii* is scattered throughout the Czech Republic^41^ and, like in Poland, it is regarded as a near-threatened (NT) species^60^. In Slovakia, the sedge is known from localities in almost the whole country; numerous data are known from basins and hilly regions in Central Slovakia and a more scattered occurrence from lowlands, basins and mountains in Western and Eastern Slovakia^12,61^. In Slovakia, the sedge is regarded as a least-concern (LC) species^62^. *Carex buekii* is widespread in SW and NE Hungary at higher altitudes^63^ and does not feature on the Hungarian Red List of vascular plants^64^. In Italy, the species is confirmed in Northern Piedmont only^7^. *Carex buekii* does not feature on the Red List of the Italian flora^65^, but as it is currently only known from a few isolated Italian sites, it should be granted the threatened species status^7^. In other European countries within the species’ range (Austria, Bulgaria, Germany, Romania, SW Russia, Serbia and Ukraine), the sedge has not been listed as threatened, except for Slovenia and Croatia where it is regarded as vulnerable (VU) and near-threatened (NT), respectively^7,66^. The occurrence of *C*. *buekii* in Bosnia and Herzegovina, Latvia and Lithuania is questionable and needs confirmation. Recently, a few old vouchers of *C*. *buekii* have been found in Greece (one record), Macedonia (two records) and Switzerland (three records)^7^. Outside Europe, the sedge grows in Azerbaijan, Georgia, Lebanon (only two records) and Turkey; the presence in Kazakhstan must be considered doubtful and needs confirmation^7^.

It should be emphasised that the occurrence of *C*. *buekii* is closely related to river floodplains, and as a river corridor plant^8^ this species should be considered as vulnerable to any human activity directed to transformation of European and Asian river valley habitats. However, taking into account the IUCN Red List Criteria, as well as its ability to colonise man-made or man-changed habitats, we propose to regard *C*. *buekii* currently as least-concern species (LC) within the centre of its occurrence.

## Supplementary information

Information about the studied area

Relevés with Carex buekii

Differences in habitat conditions at Carex buekii sites sampled

Differences in Shannon's index, evenness and number of species in phytocoenoses with Carex bueki
