# Supplementary material for: Ecology, threats and conservation status of Carex buekii (Cyperaceae) in Central Europe
Source: Sci Rep. 2019 Aug 1;9:11162. doi: 10.1038/s41598-019-47563-0 (PMC6671956; doi:10.1038/s41598-019-47563-0)
Supplement: Supplementary file 1 — Information about the studied area [file 41598_2019_47563_MOESM1_ESM.pdf]

Supplementary Table S1

Ecology, threats and conservation status of *Carex buekii* (Cyperaceae) in Central Europe

Helena Więcław[1], Kateřina Šumberová[2], Beata Bosiacka[1], Richard Hrivnák[3], Zygmunt Dajdok[4], Attila Mesterházy[5], Chiara Minuzzo[6], Edoardo Martinetto[7], Jacob Koopman[8]

[1]Institute of Marine & Environmental Sciences, University of Szczecin, Wąska 13, PL-71-415 Szczecin, Poland; [2] The Czech Academy of Sciences, Institute of Botany, Department of Vegetation Ecology, Lidická 25/27, CZ-602 00 Brno, Czech Republic; [3]Institute of Botany, Plant Science and Biodiversity Center, Slovak Academy of Sciences, Dúbravská cesta 9, SK-845 23 Bratislava, Slovakia; [4]Institute of Environmental Biology, University of Wrocław, Kanonia 6/8, PL-50-328 Wrocław, Poland; [5]Directorate of Hortobágy National Park, H-4024 Sumen utca 2, Debrecen, Hungary; [6]Regione Serramonte 10, I-10010 Andrate, Italy, [7]Earth Sciences Department, University of Turin, Via Valperga Caluso 35, I-10125 Turin, Italy, [8]ul. Kochanowskiego 27, PL-73-200 Choszczno, Poland
e-mail: helena.wieclaw@usz.edu.pl

Information about the studied area (stands of *Carex buekii*)

Expalnation: C/N – ratio between organic carbon and nitrogen concentration; org. mat. [%] – organic matter content; pH – soil pH; Ca [mg/kg] – calcium concentration; Mg [mg/kg] – magnesium concentration; P [mg/kg] – phosphorus concentration; K [mg/kg] – potassium concentration; ECe [dS/m] – electrolytic conductivity of the saturated soil extract

Locality	Coordinates	Habitat type / Shannon's diversity index	Geology / soil type/soil properties	Climate	Elevation a. s. l.	Land cover
			**Czech Republic[1,2]**			
Vltava river valley, Hluboká nad Vltavou, Munický stream I (reléve 21; see Supplementary material 2)	49°02'30.2"N, 14°26'33.0"E	high and steep artificial stream banks (probably formed by mud regularly removed from the stream) / 2.78	region: freshwater gravels and clays, in the catchment upstreams granits, gneisses, etc. / on the site loamy-clayey aluvial sediments/ org.mat=7.6; C/N=10.7; pH=5.8; Mg=94.7; P=377.1; K=672.1; Ca=1944.2; ECe=0.271.	mean annual tem. 8.0-9.0 sum of precipitation (year): 550-600 mm	373	flat fishpond basin with mosaic of tall sedge and reed beds, alluvial forests, wet grasslands and synanthropic vegetation
Vltava river valley, Hluboká nad Vltavou, Munický stream II (reléve 22)	49°02'31.4"N, 14°26'37.9"E	high and steep artificial stream banks (probably formed by mud regularly removed from the stream) / 0	region: freshwater gravels and clays, in the catchment upstreams granits, gneisses, etc. / on the site clayey aluvial sediments/ org.mat=9.4; C/N=12.5; pH=3.8; Mg=110.9; P=437.3; K=708.2; Ca=631.6; ECe=0.290.	mean annual tem. 8.0-9.0 sum of precipitation (year): 550-600 mm	371	flat fishpond basin with mosaic of tall sedge beds, wet grasslands and synanthropic vegetation

Vltava river, Hluboká nad Vltavou (reléve 23)	49°02'28.00" N, 14°26'43.7"E	high and steep artificial river bank, partly shaded by scattered trees / 0	region: freshwater gravels and clays, in the catchment upstreams granits, gneisses, etc. / on the site loamy-sandy aluvial sediments/ org.mat=10.8; C/N=14.0; pH=4.1; Mg=117.4; P=249.8; K=218.4; Ca=955.1; ECe=0.314.	mean annual tem. 8.0-9.0 sum of precipitation (year): 550-600 mm	371	flat fishpond basin with mosaic of tall sedge beds, alluvial forests, wet grasslands and tall-forb nitrophilous fringes
Svratka river, Brno-Bystrc (reléve 24)	49°13'52.2"N, 16°31'48.7"E	stony river deposit exposed to frequent water level fluctuations, partly shaded by the riverbank woody vegetation / 2.88	region and the catchment upstream gneisses, mica-schists etc., rarely also calcareous sediments / on the site sandy to loamy-sandy alluvial sediment, with admixture of large stones/ org.mat=8.6; C/N=14.3; pH=6.3; Mg=120.1; P=381.1; K=134.1; Ca=2447.7; ECe=0.561.	mean annual tem. 8.0-9.0 sum of precipitation (year): 500–550 mm	210	river valley in hilly country with mesic to alluvial forests, mesophilous grasslands and tall-forb nitrophilous fringes
Svratka river, Brno-Pisárky (reléve 25)	49°10'59.4"N, 16°34'58.5"E	lower part of high and steep river bank (artificially modified) / 2.04	region and the catchment upstream gneisses, mica-schists etc., rarely also calcareous sediments / on the site sandy-loamy to loamy-sandy alluvial sediments/ org.mat=7.7; C/N=13.7; pH=7.0; Mg=171.7; P=504.9; K=211.2; Ca=3204.9; ECe=0.537.	mean annual tem. 8.0-9.0 sum of precipitation (year): 500–550 mm	200	river valley in hilly country with mesic forests, tall nitrophilous fringes and synanthropic vegetation
Svitava river, Bílovice nad Svitavou I (reléve 26)	49°14'14.6"N, 16°39'56.7"E	high river bank formed by accumulated sediments, shaded by surrounding forest / 3.12	region and the catchment upstream: mainly calcareous clays, claystones, etc. rarely also acidic sediments / on the site: loamy-sandy to loamy alluvial sediments, mixed with organic detritus/ org.mat=7.4; C/N=14.3; pH=7.4; Mg=145.6; P=736.4; K=193.5; Ca=7589.7; ECe=0.689.	mean annual tem. 8.0-9.0 sum of precipitation (year): 500–550 mm	215	river valley in hilly country with mesic forests, riverine vegetation
Svitava river, Bílovice nad Svitavou I (reléve 27)	49°14'26.8"N, 16°40'15.1"E	high and steep river bank (artificially modified) / 3.17	region and the catchment upstream: mainly calcareous clays, claystones, etc. rarely also acidic sediments / on the site: sandy-loamy to loamy alluvial sediments/ org.mat=8.5; C/N=14.4; pH=7.4; Mg=131.5; P=717.7; K=256.2; Ca=8079.3; ECe=0.756.	mean annual tem. 8.0-9.0 sum of precipitation (year): 500–550 mm	215	river valley in hilly country with grassland, mesic forests and ruderal vegetation

Jihlava river, Letkovice near Ivančice (reléve 28)	49°05'31.6"N, 16°20'55.4"E	high river bank formed by accumulated sediments, shaded by a strip of woody vegetation / 2.19	region and the catchment upstream: acidic bedrocks such as granits and gneisses, near Ivančice also calcareous conglomerates / on the site: loamy to loamy-clayey alluvial sediments/ org.mat=6.6; C/N=11.5; pH=7.0; Mg=342.0; P=657.9; K=237.7; Ca=1896.7; ECe=0.383.	mean annual tem. 8.0-9.0 sum of precipitation (year): 500–550 mm	205	river valley in hilly country with grassland, riverine, and mesophilous to hygrophilous forest and shrub vegetation
Blanice river, Protivín (reléve 29)	49°13'09.2"N, 14°12'55.1"E	top part of high river bank (artificially modified), on the contact with regularly mown wet meadow / 2.52	region and the catchment upstream: freshwater gravels and clays, in the catchment upstreams granits, gneisses, etc. / on the site: loamy alluvial sediments/ / org.mat=9.7; C/N=12.4; pH=4.5; Mg=227.6; P=230.2; K=302.7; Ca=1140.8; ECe=0.249.	mean annual tem. 8.0-9.0 sum of precipitation (year): 550–600 mm	375	flat fishpond basin with mosaic of wet meadows, tall nitrophilous fringes and scattered shrub vegetation
Blanice river valley, Myšenec I, unnamed channel (reléve 30)	49°13'12.2"N, 14°13'02.5"E	top part of high channel bank (artificial), on the contact with regularly mown mesophilous to wet meadow, partly shaded by a strip of woody vegetation / 2.62	region and the catchment upstream: freshwater gravels and clays, in the catchment upstreams granits, gneisses, etc. / on the site: loamy alluvial sediments/ org.mat=9.2; C/N=11.2; pH=4.1; Mg=288.9; P=243.7; K=150.2; Ca=753.3; ECe=0.169.	mean annual tem. 8.0-9.0 sum of precipitation (year): 550–600 mm	375	flat fishpond basin with mosaic of wet meadows, tall nitrophilous fringes, mesic to wet forests and shrub vegetation
Blanice river, Myšenec II (reléve 31)	49°13'11.01" N, 14°13'23.6"E	high river bank (artificially modified) / 2.29	region and the catchment upstream: freshwater gravels and clays, in the catchment upstreams granits, gneisses, etc. / on the site: loamy to loamy-clayey alluvial sediments/ org.mat=12.7; C/N=12.6; pH=5.0; Mg=264.0; P=239.8; K=235.3; Ca=1755.6; ECe=0.194.	mean annual tem. 8.0-9.0 sum of precipitation (year): 550–600 mm	375	flat fishpond basin with mosaic of regularly mown wet meadows and tall nitrophilous fringes
Sázava river valley, Pohledští Dvořáci I (reléve 32)	49°36'40.5"N, 15°37'33.3"E	middle course river alluvium, partly shaded by scattered shrubs / 2.30	region and the catchment upstream: acidic bedrocks such as granits and gneisses, rarely also limestones and amphibolites / on the site: loamy alluvial sediments/ org.mat=16.4; C/N=16.6; pH=3.7; Mg=81.4; P=137.3; K=593.4; Ca=462.2; ECe=0.157.	mean annual tem. 7.0-8.0 sum of precipitation (year): 650–700 mm	415	river valley in hilly country with unmown tall nitrophilous fringes, mesic to wet shrublands and ruderal vegetation

Sázava river valley, Pohledští Dvořáci II (reléve 33)	49°36'39.1"N, 15°37'34.1"E	fishpond dam exposed to the middle course river alluvium, partly shaded by woody vegetation / 0.72	region and the catchment upstream: acidic bedrocks such as granits and gneisses, rarely also limestones and amhibolites / on the site: sandy-loamy to loamy sediments/ org.mat=10.9; C/N=14.2; pH=3.5; Mg=36.5; P=140.8; K=422.4; Ca=34.2; ECe=0.079.	mean annual tem. 7.0-8.0 sum of precipitation (year): 650–700 mm	415	river valley in hilly country with tall nitrophilous fringes, reed beds, and mesic to wet shrublands
Sázava river valley, Pohledští Dvořáci III (reléve 34)	49°36'23.1"N, 15°37'40.0"E	middle course river alluvium, partly shaded by high fishpond bank and shrub vegetation / 2.85	region and the catchment upstream: acidic bedrocks such as granits and gneisses, rarely also limestones and amhibolites / on the site: loamy-clayey alluvial sediments/ org.mat=8.0; C/N=11.7; pH=4.7; Mg=94.7; P=112.1; K=649.6; Ca=639.5; ECe=0.124.	mean annual tem. 7.0-8.0 sum of precipitation (year): 650–700 mm	415	river valley in hilly country with unmown tall nitrophilous fringes, mesophilous to hygrophilous shrublands and ruderal vegetation
Labe river valley, Tři Dvory II (reléve 35, 36)	50°01'22.3"N, 15°14'52.7"E	large shallow depression (probably former alluvial pool) with unmown vegetation in regularly mown wet alluvial meadow, partly shaded by forest margin / 1.61 / 0	region and the catchment upstream very variable, including acidic as well as basic bedrocks (e.g. calcareous and noncalcareous sandstones and conglomerates, phyllites) / on the site loamy-clayey alluvial sediments / org.mat=13.7; C/N=11.8; pH=5.1; Mg=86.7; P=145.2; K=493.8; Ca=3212.9; ECe=0.116 / org.mat=13.4; C/N=12.0; pH=5.3; Mg=90.1; P=242.0; K=770.9; Ca=3089.6; ECe=0.183.	mean annual tem. 9.0–10.0 sum of precipitation (year): 550–600 mm	195	broad river floodplain with the mosaic of mown mesophilous to wet meadows, hard-wooded alluvial forests and unmown nitrophilous fringes
Labe river valley, Kolín (reléves 37)	50°01'22.2"N, 15°13'52.00"E	on the top of high bank of river oxbow, partly shaded by forest / 2.62	region and the catchment upstream very variable, including acidic as well as basic bedrocks (e.g. calcareous and noncalcareous sandstones and conglomerates, phyllites) / on the site loamy-sandy alluvial sediments/ org.mat=9.2; C/N=14.0; pH=7.2; Mg=86.8; P=303.9; K=342.1; Ca=6657.3; ECe=0.236.	mean annual tem. 9.0–10.0 sum of precipitation (year): 550–600 mm	195	broad river floodplain with the mosaic of arable field, remnants of mesophilous grasslands and hard-wooded alluvial forests, and unmown nitrophilous fringes

Orlice river valley, Hradec Králové-Malšovice I (reléve 38)	50°12'28.6"N, 15°51'16.5"E	shallow depression on the margin of mown mesophilous meadow, partly shaded by forest / 1.61	region and the catchment upstream: mainly calcareous sediments, e.g. marls, argillites, conglomerates and sandstones, more rarely acidic bedrocs, e.g. granits and gneisses / on the site sandy-loamy to clayey-loamy alluvial sediments/ org.mat=7.8; C/N=12.7; pH=4.8; Mg=47.9; P=187.9; K=310.8; Ca=2168.0; ECe=0.093.	mean annual tem. 8.0–9.0 sum of precipitation (year): 600–650 mm	225	mesophilous to wet grasslands and remnants of hard-wooded alluvial forests
Orlice river valley, Hradec Králové-Malšovice II (reléve 39)	50°12'30.6"N, 15°51'30.2"E	bank of the river arm (now adjusted for fish farming use), shaded by forest / 3.12	region and the catchment upstream: mainly calcareous sediments, e.g. marls, argillites, conglomerates and sandstones, more rarely acidic bedrocs, e.g. granits and gneisses / on the site sandy-loamy to loamy alluvial sediments / org.mat=8.3; C/N=12.1; pH=5.0; Mg=50.0; P=96.8; K=175.9; Ca=1966.7; ECe=0.136.	mean annual tem. 8.0–9.0 sum of precipitation (year): 600–650 mm	225	reed beds, unmown nitrophilous fringes and remnants of hard-wooded alluvial forests
Orlice river valley, Hradec Králové-Malšovice III (reléve 40)	50°12'35.5"N, 15°51'47.3"E	margin of abandoned wet meadow at a river arm / 1.61	region and the catchment upstream: mainly calcareous sediments, e.g. marls, argillites, conglomerates and sandstones, more rarely acidic bedrocs, e.g. granits and gneisses / on the site loamy-sandy to loamy alluvial sediments / org.mat=9.0; C/N=11.2; pH=4.5; Mg=78.4; P=126.0; K=114.0; Ca=1608.6; ECe=0.106.	mean annual tem. 8.0–9.0 sum of precipitation (year): 600–650 mm	230	mesophilous to wet alluvial meadows, unmown nitrophilous fringes and remnants of hard-wooded alluvial forests
**Hungary**[3]						
Kerca rivulet, Kercaszomor (reléve 62)	46°46′54″N, 16°19'51"E	banks of Kerca-rivulet, in alder forest / 4.05	aluvial sands, gravels and muds/ org.mat=13.9; C/N=11.4; pH=3.9; Mg=65.4; P=63.4; K=195.9; Ca=463.3; ECe=0.178.	mean annual tem. 9.0-10.0 sum of precipitation (year): 800-850 mm	230	alder forest
Pinka river, Felsőcsatár (reléve 63)	47°12'33"N, 16°25'55"E	banks of Pinka-river in willow forest / 2.97	aluvial sands, gravels and muds / org.mat=8.3; C/N=10.2; pH=5.9; Mg=177.3; P=127.3; K=73.9; Ca=2330.4; ECe=0.267.	mean annual tem. 9.0-10.0 sum of precipitation (year): 800-850 mm	245	willow forest

Huszászi stream, Csörötnek (reléve 64)	46°56'12"N, 16°22'40"E	in alder forest near Huszászi-stream / 3.84	aluvial sands, gravels and muds / org.mat=7.2; C/N=11.9; pH=3.6; Mg=74.3; P=31.0; K=67.5; Ca=468.6; ECe=0.142.	mean annual tem. 9.0-10.0 sum of precipitation (year): 800-850 mm	230	alder forest
Zala stream, Őriszentpéter (reléve 66)	46°50'38"N, 16°23'51"E	banks of Zala-stream in willow forest / 3.21	aluvial sands, gravels and muds / org.mat=11.1; C/N=9.8; pH=5.6; Mg=223.5; P=110.7; K=118.0; Ca=2868.5; ECe=0.152.	mean annual tem. 9.0-10.0 sum of precipitation (year): 800-850 mm	230	willow forest
Rába river, Körmend (reléve 65)	46°59'45"N, 16°35'56"E	banks of a small oxbow / 3.22	aluvial sands, gravels and muds / org.mat=6.6; C/N=9.9; pH=5.9; Mg=78.3; P=31.0; K=53.8; Ca=2016.7; ECe=0.225.	mean annual tem. 9.0-10.0 sum of precipitation (year): 800-850 mm	185	edge of floodplain meadows
Apátistvánfalvai stream, Apátistvánfalva (reléves 67, 68)	46°54'47"N, 16°17'45"E	in wet alder forest near Apátistvánfalvi-stream / 3.71/ 2.81	aluvial sands, gravels and muds / org.mat=7.1; C/N=11.1; pH=6.0; Mg=132.3; P=82.0; K=60.2; Ca=1801.6; ECe=0.097 / org.mat=22.2; C/N=11.9; pH=4.0; Mg=169.6; P=125.6; K=141.3; Ca=1886.5; ECe=0.707.	mean annual tem. 9.0-10.0 sum of precipitation (year): 800-850 mm	255	alder forest
Tarna rivulet, Kompolt (reléve 69)	47°45'10"N, 20°15'05"E	edge of a haymeadow 100 m of Tarna-rivulet / 3.51	aluvial sands, gravels and muds / org.mat=9.2; C/N=10.9; pH=5.9; Mg=105.6; P=256.4; K=466.5; Ca=3129.7; ECe=0.167.	mean annual tem. 9.0-10.0 sum of precipitation (year): 500-550 mm	115	edge of semidry hay meadow, near a crop field
Kerka rivulet, Kerkáskápolna (reléve 70)	46°46'52"N, 16°25'06"E	banks of an old riverbed in an alder forest / 3.84	aluvial sands, gravels and muds / org.mat=8.6; C/N=11.1; pH=4.7; Mg=126.9; P=181.8; K=232.9; Ca=1270.8; ECe=0.428.	mean annual tem. 9.0-10.0 sum of precipitation (year): 800-850 mm	208	alder forest
Szentgyörgyvölgyi stream, Szentgyörgyvölgy (reléve 71)	46°43'37"N, 16°23'57"E	on a hillside, near near a spring / 4.25	aluvial sands, gravels and muds / org.mat=14.7; C/N=12.9; pH=5.5; Mg=116.8; P=149.1; K=93.1; Ca=1907.8; ECe=0.248.	mean annual tem. 9.0-10.0 sum of precipitation (year): 800-850 mm	194	grazed mezophilous meadow near a spring
Kerka rivulet, Bajánsenye (reléve 72)	46°47'54"N, 16°22'51"E	banks of Kerka-rivulet, in a wet willow forest / 3.14	aluvial sands, gravels and muds / org.mat=11.3; C/N=12.2; pH=3.3; Mg=76.2; P=58.0; K=85.9; Ca=86.5; ECe=0.087.	mean annual tem. 9.0-10.0 sum of precipitation (year): 800-850 mm	225	willow forest

Hór stream, Répáshuta (reléve 73)	48°01'54"N, 20°33'18"E	former hay meadow, which was abandoned and covered with *Magnocaricion* stands / 2.22	aluvial sands, gravels and muds / org.mat=14.4; C/N=14.0; pH=6.8; Mg=751.4; P=330.9; K=298.7; Ca=14455.0; ECe=0.944.	mean annual tem. 5.0-6.0 sum of precipitation (year): 650-700 mm	370	abandoned hay meadow
Bán stream, Bélapátfalva (reléve 74)	48°01'15"N, 20°19'44"E	former hay meadow, which was abandoned and covered with *Magnocaricion* stands / 2.85	aluvial sands, gravels and muds / org.mat=11.2; C/N=12.0; pH=7.3; Mg=239.3; P=325.3; K=638.4; Ca=19296.8; ECe=0.364.	mean annual tem. 7.0-8.0 sum of precipitation (year): 600-650 mm	265	abandoned hay meadow
Csermely stream, Lénárddaróc (reléve 75)	48°09'09"N, 20°22'54"E	former hay meadow, which was abandoned and covered with *Magnocaricion* stands / 3.19	aluvial sands, gravels and muds / org.mat=12.0; C/N=12.4; pH=7.3; Mg=538.3; P=425.1; K=228.1; Ca=33633.3; ECe=0.406.	mean annual tem. 7.0-8.0 sum of precipitation (year): 600-650 mm	270	abandoned hay meadow
Eger stream, Mikófalva (reléve 76)	48°04'05"N, 20°18'55"E	former hay meadow, which was abandoned and covered with *Magnocaricion* stands / 3.57	aluvial sands, gravels and muds / org.mat=8.2; C/N=12.5; pH=7.3; Mg=861.1; P=250.7; K=291.5; Ca=14736.9; ECe=0.318.	mean annual tem. 7.0-8.0 sum of precipitation (year): 600-650 mm	284	abandoned hay meadow
Bán stream, Nagyvisnyó reléve 77)	48°07'46"N, 20°24'11"E	banks of Bán-stream in an alder forest / 3.98	aluvial sands, gravels and muds / org.mat=11.2; C/N=10.7; pH=6.4; Mg=182.9; P=178.3; K=215.2; Ca=3376.6; ECe=0.482.	mean annual tem. 7.0-8.0 sum of precipitation (year): 600-650 mm	300	alder forest near a pasture
Ipoly river, Ipolyvece I (reléve 78)	48°04'11"N, 19°07'21"E	banks of Ipoly river in willow forest / 3.61	aluvial sands, gravels and muds / org.mat=9.3; C/N=11.6; pH=5.1; Mg=191.3; P=312.6; K=262.6; Ca=1864.9; ECe=0.200.	mean annual tem. 9.0-10.0 sum of precipitation (year): 500-550 mm	130	edge of willow forest near an alluvial hay meadow
Ipoly river, Ipolyvece II (reléve 79)	48°03'57"N, 19°06'43"E	banks of an old oxbow, near a hay meadow / 3.69	aluvial sands, gravels and muds / org.mat=8.0; C/N=12.5; pH=5.7; Mg=104.6; P=303.5; K=248.9; Ca=2205.5; ECe=0.117.	mean annual tem. 9.0-10.0 sum of precipitation (year): 500-550 mm	130	edge of floodplain meadows
Ipoly river, Drégelypalánk (reléve 80)	48°03'57"N, 19°02'48"E	banks of Ipoly river in willow forest / 3.21	aluvial sands, gravels and muds / org.mat=10.3; C/N=10.8; pH=6.4; Mg=219.8; P=327.0; K=220.0; Ca=3093.2; ECe=0.207.	mean annual tem. 9.0-10.0 sum of precipitation (year): 500-550 mm	126	willow forest near an alluvial hay meadow
**Italy[4]**						

Malone river valley, Lombardore (reléve 81)	45°14'40"N, 7°43'59"E	banks of a small oxbow-type of environment / 3.45	aluvial sands, gravels and muds / org.mat=4.0; C/N=11.4; pH=5.4; Mg=102.6; P=106.4; K=41.8; Ca=396.7; ECe=0.045.	mean annual tem. 12.1 sum of precipitation (year): 848 mm	229	tall-sedge communities of the old river banks (*Magnocaricion*), with bushes and separate trees around (willows and alders)
Malone river valley, Rivarossa (reléve 82)	45°15'16"N, 7°43'49"E	old floodplain (no more flooded) of the Malone river / 3.40	river alluvial soils / org.mat=9.9; C/N=10.3; pH=4.8; Mg=103.4; P=100.7; K=94.0; Ca=1146.6; ECe=0.124.	mean annual tem. 11.9-12.0 sum of precipitation (year): 852 mm	235	tall-sedge communities of long time abandoned field or meadow (*Magnocaricion*), with a lot of woodland and *Populus* plantations around
Malone river valley, Front (reléve 83)	45°16'42"N, 7°40'49"E	old floodplain (no more flooded) of the Malone river / 2.98	river alluvial soils / org.mat=9.4; C/N=11.4; pH=4.2; Mg=109.4; P=63.7; K=183.1; Ca=693.7; ECe=0.095.	mean annual tem. 11.7 sum of precipitation (year): 857 mm	261	tall-sedge communities of long time abandoned field or meadow (*Magnocaricion*), with bushes and separate trees, within a mosaic of floodplain meadows and crop fields
Malone river valley, Front (reléve 84)	45°16'23"N, 7°41'38"E	old floodplain (no more flooded) of the Malone river / 3.51	river alluvial soils / org.mat=14.8; C/N=12.5; pH=5.6; Mg=156.1; P=526.3; K=182.3; Ca=1823.6; ECe=0.214.	mean annual tem. 11.7 sum of precipitation (year): 857 mm	253	tall-sedge communities of long time abandoned field or meadow (*Magnocaricion*), with bushes and separate trees around, within a mosaic of floodplain meadows and crop fields
			**Poland**[5,6,7]			
Odra river valley, Wrocław-Swojec (reléve 1)	51°06'49.8"N, 17°06'40.4"E	banks of the navigation canal on the odra river floodplain / 3.23	river alluvial soils / org.mat=7.0; C/N=13.2; pH=5.1; Mg=39.2; P=201.0; K=493.8; Ca=1111.9; ECe=0.203.	mean annual temp. 8.0-8.5; sum of precipitation (year): 550-600 mm	113	regenerative managed vegetation, with bushes and separate trees
Odra river valley, Wrocław-Wojnów (Strachocin) (reléve 2)	51°05'46.5"N, 17°08'07.9"E	small depression on the Odra river floodplain / 3.26	river alluvial soils / org.mat=9.7; C/N=12.1; pH=4.6; Mg=69.7; P=266.0; K=372.6; Ca=931.2; ECe=0.194.	mean annual temp. 8.0-8.5; sum of precipitation (year): 550-600 mm	118	floodplain meadows (*Cnidion*), currently not mown

Odra river valley, Janowice (Jeszkowice-Jakubowice) (reléve 3)	51°02'35.2"N, 17°12'04.3"E	small depression on the Odra river floodplain / 2.73	river alluvial soils / org.mat=18.8; C/N=18.0; pH=4.5; Mg=75.2; P=512.3; K=434.4; Ca=1326.9; ECe=0.274.	mean annual temp. 8.0-8.5; sum of precipitation (year): 550-600 mm	120	mosaic of floodplain meadows (*Cnidion*) and crop fields
Odra river valley, Janowice (Jeszkowice) (reléve 4)	51°02'43.0"N, 17°12'25.7"E	banks of Odra old river bed / 2.78	river alluvial soils / org.mat=14.0; C/N=15.2; pH=4.3; Mg=88.5; P=376.3; K=363.8; Ca=957.8; ECe=0.208.	mean annual temp. 8.0-8.5; sum of precipitation (year): 550-600 mm	120	mosaic of floodplain meadows (*Cnidion*) and crop fields
Odra river valley, Siechnice I (reléve 5)	51°02'25.3"N, 17°11'43.2"E	floodplain of the Odra river / 3.09	river alluvial soils / org.mat=11.9; C/N=14.4; pH=4.9; Mg=91.9; P=256.4; K=248.9; Ca=1689.0; ECe=0.173.	mean annual temp. 8.0-8.5; sum of precipitation (year): 550-600 mm	122	transition zone between riverside elm carrs and floodplain meadows (*Cnidion*)
Odra river valley, Siechnice II (reléve 6)	51°02'17.1"N, 17°11'32.1"E	floodplain of the Odra river / 4.24	river alluvial soils / org.mat=11.6; C/N=12.1; pH=4.7; Mg=103.1; P=110.3; K=265.0; Ca=1776.9; ECe=0.178.	mean annual temp. 8.0-8.5; sum of precipitation (year): 550-600 mm	121	mosaic of floodplain meadows (*Cnidion*) and tall-sedge communities
Kaczawa river valley, Kwiatkowice (reléve 7)	51°17'12.5"N, 16°25'12.0"E	floodplain of the Kaczawa and Odra rivers / 3.11	river alluvial soils / org.mat=13.3; C/N=11.5; pH=4.8; Mg=94.0; P=524.1; K=293.1; Ca=1341.3; ECe=0.237.	mean annual temp. 8.0-8.5; sum of precipitation (year): 500-550 mm	95	mosaic of floodplain meadows (*Cnidion*) and tall-sedge communities
Odra river valley, Gosławice (reléve 8)	51°13'21.2"N, 16°49'37.6"E	artificial railway embankment in the floodplain of the Odra river / 3.46	artificial substratum / org.mat=6.4; C/N=17.3; pH=6.0; Mg=53.6; P=336.2; K=308.4; Ca=1321.3; ECe=0.123.	mean annual temp. 8.0-8.5; sum of precipitation (year): 550-600 mm	108	sedge communit on the edge of the railway embankment
Odra river valley, Warzyna (reléve 9)	51°15'18.5"N, 16°46'44.6"E	floodplain of the Odra river / 3.16	river alluvial soils / org.mat=5.7; C/N=12.0; pH=4.8; Mg=44.4; P=165.7; K=765.3; Ca=514.4; ECe=0.156.	mean annual temp. 8.0-8.5; sum of precipitation (year): 550-600 mm	106	mosaic of floodplain meadows *(Alopecurion*) and sege communities (*Magnocaricion*)
Bystrzyca river valley, Wrocław - Jarnołtów (reléve 10)	51°07'29.9"N, 16°50'30.9"E	small depression on the Bystrzyca river floodplain / 2.64	river alluvial soils / org.mat=13.5; C/N=16.7; pH=5.3; Mg=97.8; P=378.4; K=384.6; Ca=1522.9; ECe=0.214.	mean annual temp. 8.0-8.5; sum of precipitation (year): 550-600 mm	115	mosaic of floodplain meadows (*Alopecurio*n) and sege communities (*Magnocaricion*)

Bystrzyca river valley, Wrocław – Ratyń (reléve 11)	51°07'30.8"N, 16°50'40.2"E	banks of the Bystrzyca old river bed / 2.85	river alluvial soils / org.mat=15.5; C/N=18.5; pH=5.1; Mg=72.1; P=985.4; K=454.5; Ca=915.4; ECe=0.205.	mean annual temp. 8.0-8.5; sum of precipitation (year): 550-600 mm	116	mosaic of floodplain meadows (*Alopecurion*) and sege communities (*Magnocaricion*)
Odra river valley, Kopanie I (reléve 12)	50°48'47.3"N, 17°38'08.1"E	floodplain of the Odra river / 2.72	river alluvial soils / org.mat=11.5; C/N=11.9; pH=4.5; Mg=104.9; P=211.0; K=272.2; Ca=1301.5; ECe=0.250.	mean annual temp. 8.0-8.5; sum of precipitation (year): 600-650 mm	139	*Populs* plantantion
Odra river valley, Kopanie II (reléve 13)	50°48'49.5"N, 17°38'09.7"E	floodplain of the Odra river / 3.01	river alluvial soils / org.mat=12.6; C/N=12.1; pH=5.7; Mg=91.0; P=782.6; K=285.1; Ca=2404.2; ECe=0.315.	mean annual temp. 8.0-8.5; sum of precipitation (year): 600-650 mm	138	tall-sedge communiteis of the old river banks (*Magnocaricion*)
Odra river valley, Kruszyna (reléve 14)	50°50'28.4"N, 17°32'42.7"E	floodbank the in floodplain of the Odra river / 3.21	artificial substratum / org.mat=5.3; C/N=17.3; pH=4.3; Mg=48.2; P=120.3; K=290.7; Ca=471.8; ECe=0.116.	mean annual temp. 8.0-8.5; sum of precipitation (year): 600-650 mm	136	mosaic of sedge and meadow communities of the floodbank
Odra river valley, Kotowice – Czernica (reléve 15)	51°02'43.5"N, 17°13'18.1"E	floodplain of the Odra river / 2.89	river alluvial soils / org.mat=13.3; C/N=23.3; pH=5.1; Mg=54.3; P=898.6; K=142.9; Ca=901.8; ECe=0.151.	mean annual temp. 8.0-8.5; sum of precipitation (year): 550-600 mm	125	tall-sedge communities of the banks of Odra tributary (*Magnocaricion*)
Oława river valley, Wrocław - Mokry Dwór (reléve 16)	51°04'11.0"N 17°08'21.3"E	floodplain of the Oława river / 3.16	river alluvial soils / org.mat=14.8; C/N=11.6; pH=4.5; Mg=116.4; P=220.6; K=333.2; Ca=2150.7; ECe=0.234.	mean annual temp. 8.0-8.5; sum of precipitation (year): 550-600 mm	118	mosaic of floodplain meadows (Cnidion) and sedge communities (*Magnocaricion*)
Odra river valley, Trestno (reléve 17)	51°04'32.1"N, 17°09'09.5"E	floodplain of the Odra river / 3.83	river alluvial soils / org.mat=8.9; C/N=11.6; pH=4.8; Mg=123.4; P=246.3; K=459.3; Ca=1644.6; ECe=0.211.	mean annual temp. 8.0-8.5; sum of precipitation (year): 550-600 mm	119	tall-sedge communiteis of the Odra river floodplain (*Magnocaricion*)
Oława river valley, Wrocław – Światniki (reléve 18)	51°05'40.0"N, 17°06'31.1"E	floodplain of the Oława river / 3.97	river alluvial soils / org.mat=14.8; C/N=12.6; pH=5.2; Mg=98.3; P=333.1; K=485.8; Ca=2500.6; ECe=0.271.	mean annual temp. 8.0-8.5; sum of precipitation (year): 550-600 mm	117	tall-sedge communiteis of the Oława river floodplain (*Magnocaricion*)
Oława river valley, Wrocław - Nowy Dom (reléve 19)	51°05'31.8"N, 17°06'10.2"E	banks of the Oława river near its outlet to Odra river / 3.31	river alluvial soils / org.mat=13.0; C/N=12.5; pH=5.7; Mg=86.4; P=230.6; K=950.8; Ca=2654.7; ECe=0.261.	mean annual temp. 8.0-8.5; sum of precipitation (year): 550-600 mm	118	tall-sedge communiteis of the Oława river banks (*Magnocaricion*)

Odra river valley, Stary Otok (reléve 20)	50°58'41.7"N, 17°19'56.4"E	banks of the old river bed of Odra river / 3.44	river alluvial soils / org.mat=3.9; C/N=13.5; pH=3.7; Mg=12.3; P=88.5; K=101.2; Ca=462.0; ECe=0.048.	mean annual temp. 8.0-8.5; sum of precipitation (year): 550-600 mm	126	tall-sedge community of the sandy banks of old river bed
			**Slovakia[8]**			
Veľká Lúka, sw. from the village (reléve 41)	48°37'32.4"N, 19°09'33.5"E	bank of the canal near road / 3.22	clays with lignite, sands, gravels / org.mat=11.6; C/N=12.4; pH=7.0; Mg=296.0; P=307.8; K=534.8; Ca=6679.9; ECe=0.320.	mean annual tem. 7.0-8.0 sum of precipitation (year): 600-700 mm	304	crop fields and pastures
Lukavica, n. margin of the village (reléve 42)	48°38'39.8"N, 19°12'32.4"E	bank of the stream / 1.94	clays with lignite, sands, gravels / org.mat=6.1; C/N=12.2; pH=6.8; Mg=68.1; P=100.7; K=640.8; Ca=1948.2; ECe=0.141.	mean annual tem. 6.0-7.0 sum of precipitation (year): 700-800 mm	344	floodplain meadows and shrubs
Slovenská Ľupča, sw. from the village (reléve 43)	48°45'15.1"N, 19°15'33.3"E	terrain depression on the Hron river floodplain / 3.32	dark-gray limestones / org.mat=8.7; C/N=14.4; pH=7.4; Mg=751.5; P=266.8; K=369.4; Ca=8536.0; ECe=0.150.	mean annual tem. 7.0-8.0 sum of precipitation (year): 700-800 mm	371	artificial surfaces and floodplain meadows
Valaská, se. from the village (reléve 44)	48°48'44.1"N, 19°35'26.7"E	bank of the river / 2.66	varied shales, sandstones and dolomites / org.mat=5.1; C/N=15.9; pH=7.4; Mg=333.5; P=396.8; K=133.3; Ca=4512.7; ECe=0.091.	mean annual tem. 7.0-8.0 sum of precipitation (year): 700-800 mm	480	floodplain meadows, riparian shrubs and forests
Pitelová, Čierne Zeme settlement (reléve 45)	48°35'29.8"N, 18°55'24.2"E	bank of the canal near road / 1.48	pyroxene and hornblende-pyroxene andesites / org.mat=9.2; C/N=13.5; pH=6.1; Mg=102.1; P=301.7; K=974.0; Ca=2197.9; ECe=0.184.	mean annual tem. 8.0-9.0 sum of precipitation (year): 600-700 mm	264	pastures and artificial surfaces
Turček, nw. from the village (reléve 46)	48°46'34.2"N, 18°53'09.9"E	terrain depression on the Turiec river floodplain / 1.74	basaltic and pyroxene andesites / org.mat=15.8; C/N=13.2; pH=5.1; Mg=60.3; P=317.8; K=218.4; Ca=2144.7; ECe=0.635.	mean annual tem. 5.0-6.0 sum of precipitation (year): 900-1000 mm	616	marshes, riparian shrubs and forests
Moškovec, sse. from the village (reléve 47)	48°56'23.4"N, 18°49'47.5"E	bank of the Turiec river / 2.11	gray and varied clays, sands, gravels / conglomerates, lignite seams, freshwater limestones, rhyolite and andesite tuffs / org.mat=12.9; C/N=14.2; pH=7.3; Mg=2056.0; P=379.3; K=220.0; Ca=20943.1; ECe=0.240.	mean annual tem. 7.0-8.0 sum of precipitation (year): 700-800 mm	445	floodplain meadows

Zvolen, Môťová (reléve 48)	48°33'21.1"N, 19°11'28.9"E	bank of the Slatina river / 1.81	pyroxene and hornblende-pyroxene andesites / org.mat=8.3; C/N=11.3; pH=4.6; Mg=74.3; P=274.7; K=360.5; Ca=1743.4; ECe=0.116.	mean annual tem. 8.0-9.0 sum of precipitation (year): 600-700 mm	307	floodplain meadows, riparian shrubs, forests
Pstruša, near railway station (reléve 49)	48°33'08.9"N, 19°18'34.9"E	terrain depression on the Slatina river floodplain / 2.16	clays with lignite, sands, gravels / org.mat=8.3; C/N=11.1; pH=4.6; Mg=41.9; P=224.5; K=611.1; Ca=1137.5; ECe=0.096.	mean annual tem. 7.0-8.0 sum of precipitation (year): 600-700 mm	316	floodplain meadows, shrubs and artificial surfaces
Tomášovce, s. from the village (reléve 50)	48°24'52.8"N, 19°35'31.3"E	bank of cannalized stream / 1.93	varied kaolinite clays, sands, gravels, rare lignite seams / org.mat=8.3; C/N=11.8; pH=6.2; Mg=145.8; P=269.0; K=696.2; Ca=1807.3; ECe=0.140.	mean annual tem. 8.0-9.0 sum of precipitation (year): 550-600 mm	220	floodplian meadows and shrubs
Breznička, Červeň settlement (reléve 51)	48°24'42.6"N, 19°44'10.7"E	bank of the Ipeľ river / 4.04	metamorphosed sandstones and conglomerates, phyllites, mafic volcanites / org.mat=2.6; C/N=14.4; pH=6.0; Mg=38.8; P=323.1; K=119.6; Ca=731.0; ECe=0.064.	mean annual tem. 8.0-9.0 sum of precipitation (year): 600-700 mm	210	floodplain meadows, marshes, shrubs and forests
Vyšný Skálnik, w. from the village (reléve 52)	48°27'55.0"N, 19°57'36.4"E	terrain depression on the Rimava river floodplain / 2.43	gray calcareous siltstones / org.mat=14.4; C/N=12.8; pH=6.6; Mg=156.3; P=300.0; K=382.2; Ca=3298.6; ECe=0.258.	mean annual tem. 8.0-9.0 sum of precipitation (year): 600-700 mm	232	crop fields, floodplain meadows and artificial surfaces
Žihľava, n. from the village (reléve 53)	48°13'38.5"N, 19°25'05.6"E	terrain depression on the Stará rieka stream floodplain / 2.46	gray claystones, sands, coal seams, carbonaceous clays / org.mat=7.7; C/N=11.3; pH=6.0; Mg=83.9; P=127.7; K=411.1; Ca=1925.8; ECe=0.155.	mean annual tem. 9.0-10.0 sum of precipitation (year): 550-600 mm	203	floodplain meadows, artificial surfaces and shrubs
Dolná Strehová, n. from the village (reléve 54)	48°16'13.0"N, 19°26'52.5"E	terrain depression on the Tisovník stream floodplain / 3.28	pyroxene and hornblende-pyroxene andesites / org.mat=5.7; C/N=10.2; pH=6.4; Mg=110.6; P=102.5; K=104.4; Ca=1710.7; ECe=0.134.	mean annual tem. 9.0-10.0 sum of precipitation (year): 550-600 mm	199	marshes, floodplain meadows and crop fields
Jalšovník, s. margin of the village (reléve 55)	48°18'30.1"N, 19°06'05.0E	bank of the canal near road / 2.24	pyroxene and hornblende-pyroxene andesites / org.mat=10.3; C/N=13.2; pH=5.8; Mg=108.9; P=150.9; K=197.5; Ca=2090.9; ECe=0.201.	mean annual tem. 8.0-9.0 sum of precipitation (year): 550-600 mm	329	floodplain meadows, artificial surfaces and shrubs

Krupina, s. margin of the town (reléve 56)	48°19'30.4"N, 19°03'43.6"E	bank of the Krupinica river / 3.78	pyroxene and hornblende-pyroxene andesites / org.mat=6.8; C/N=11.6; pH=5.9; Mg=92.5; P=129.5; K=305.1; Ca=1932.6; ECe=0.129.	mean annual tem. 8.0-9.0 sum of precipitation (year): 550-600 mm	251	crop fields, artificial surfaces and riparian shrubs
Rykynčice, n. from the village (reléve 57)	48°13'01.5"N, 18°58'24.7"E	terrain depression on the Krupinica stream floodplain / 3.18	gray calcareous siltstones, claystones, sandstones, conglomerates, algal limestones, rhyolite and andezite tuffs / org.mat=7.2; C/N=12.4; pH=5.8; Mg=79.4; P=721.6; K=259.4; Ca=1663.7; ECe=0.156.	mean annual tem. 8.0-9.0 sum of precipitation (year): 550-600 mm	161	floodplain meadows and shrubs
Šávoľ, wsw. from the village (reléve 58)	48°18'04.9"N, 19°48'50.6"E	bank of the canalized Suchá stream / 1.92	gray calcareous siltstones/ org.mat=8.1; C/N=12.1; pH=7.3; Mg=240.0; P=392.8; K=545.2; Ca=4472.6; ECe=0.222.	mean annual tem. 8.0-9.0 sum of precipitation (year): 550-600 mm	188	crop fields and artificial surfaces
Petrovce, ne. from the village (reléve 59)	48°11'37.5"N, 20°01'58.4"E	bank of the Mačací potok stream / 3.53	siltstones, claystones, sandstones, tuffites, varied and coal clays, coal, conglomerates, organodetritic limestones / org.mat=5.5; C/N=12.6; pH=7.6; Mg=493.0; P=454.7; K=676.9; Ca=9106.9; ECe=0.190.	mean annual tem. 7.0-8.0 sum of precipitation (year): 550-600 mm	225	floodplain meadows and shrubs
Janice, n. from the village (reléve 60)	48°17'18.4"N, 20°12'51.6"E	bank of the canal near road / 1.72	siltstones, claystones, sandstones, tuffites, varied and coal clays, coal, conglomerates, organodetritic limestones / org.mat=8.5; C/N=10.0; pH=6.9; Mg=205.2; P=507.1; K=522.8; Ca=3020.4; ECe=0.274.	mean annual tem. 8.0-9.0 sum of precipitation (year): 550-600 mm	164	crop fields
Hrušovo, n. from the village (reléve 61)	48°31'11.4"N, 20°02'49.0"E	terrain depression on the Blh stream floodplain / 3.66	dark limestones, recrystallized and cherty limestones, dark schistose limestones / org.mat=8.5; C/N=11.7; pH=6.9; Mg=189.9; P=220.6; K=157.4; Ca=2495.7; ECe=0.148.	mean annual tem. 7.0-8.0 sum of precipitation (year): 600-700 mm	251	floodplain meadows and shrubs

[1] Geological maps of the Czech Geological Service (http://www.geology.cz/extranet/mapy/mapy-online/mapove-aplikace)

[2] Tolasz R (ed.) (2007) Climate atlas of Czechia (in English and Czech). Český hydrometeorologický ústav and Univerzita Palackého v Olomouci, Praha & Olomouc.

[3]https://www.metnet.hu/

[4]Dati climatici sulle città del mondo - Climate-Data.org (https://it.climate-data.org/)

[5]Bac-Bronowicz J (1997) Opady atmosferyczne (1951-1980). In: Pawlak W (ed) Atlas Śląska Dolnego i Opolskiego. Uniwersytet Wrocławski, Pracownia Atlasu Dolnego Śląska, Wrocław, p. 43.

[6]Piasecki J (1997) Temperatura powietrza (1951-1980). In: Pawlak W (ed) Atlas Śląska Dolnego i Opolskiego. Uniwersytet Wrocławski, Pracownia Atlasu Dolnego Śląska, Wrocław, p. 47.

[7]Borkowski J, Wojniak R (1997) Rodzaje i gatunki gleb. In: Pawlak W (ed) Atlas Śląska Dolnego i Opolskiego. Uniwersytet Wrocławski, Pracownia Atlasu Dolnego Śląska, Wrocław, pp. 31–32.

[8]Miklos L (ed) (2002) Atlas krajiny Slovenskej republiky (1. vydanie). MŽP SR, Bratislava and SAŽP, Banská Bystrica, 344 pp.
