# Supplementary material for: Ecology, threats and conservation status of Carex buekii (Cyperaceae) in Central Europe
Source: Sci Rep. 2019 Aug 1;9:11162. doi: 10.1038/s41598-019-47563-0 (PMC6671956; doi:10.1038/s41598-019-47563-0)
Supplement: Supplementary file 2 — Relevés with Carex buekii [file 41598_2019_47563_MOESM2_ESM.pdf]

Supplementary Table S2

Ecology, threats and conservation status of *Carex buekii* (Cyperaceae) in Central Europe

Helena Więcław[1], Kateřina Šumberová[2], Beata Bosiacka[1], Richard Hrivnák[3], Zygmunt Dajdok[4], Attila Mesterházy[5], Chiara Minuzzo[6], Edoardo Martinetto[7], Jacob Koopman[8]

[1]Institute of Marine & Environmental Sciences, University of Szczecin, Wąska 13, PL-71-415 Szczecin, Poland; [2] The Czech Academy of Sciences, Institute of Botany, Department of Vegetation Ecology, Lidická 25/27, CZ-602 00 Brno, Czech Republic; [3]Institute of Botany, Plant Science and Biodiversity Center, Slovak Academy of Sciences, Dúbravská cesta 9, SK-845 23 Bratislava, Slovakia; [4]Institute of Environmental Biology, University of Wrocław, Kanonia 6/8, PL-50-328 Wrocław, Poland; [5]Directorate of Hortobágy National Park, H-4024 Sumen utca 2, Debrecen, Hungary; [6]Regione Serramonte 10, I-10010 Andrate, Italy, [7]Earth Sciences Department, University of Turin, Via Valperga Caluso 35, I-10125 Turin, Italy, [8]ul. Kochanowskiego 27, PL-73-200 Choszczno, Poland

Relevés with *Carex buekii* (bold font - species found in more than 2 samples)

Detailed informations about the *Carex buekii* stands are in Supplementary material 1

Releve no.	1	2	3	4	5	6	7	8	9	10	11	12	13	14	15	16	17	18	19	20	21	22	23	24	25	26	27	28	29	30	31	32	33	34	35	36	37	38	39	40	41	42	43	44	45	46	47	48	49	50	51	52	53	54	55	56	57	58	59	60	61	62	63	64	65	66	67	68	69	70	71	72	73	74	75	76	77	78	79	80	81	82	83	84
Country	Polnad																				Czech Republic																				Slovakia																					Hungary																			Italy			
Acer negundoA	-	-	-	-	-	-	-	-	-	-	-	-	-	-	-	-	-	-	-	-	-	-	-	-	-	-	-	-	-	-	-	-	-	-	-	-	-	-	-	-	-	-	-	-	-	-	-	-	-	-	-	-	-	-	-	-	-	-	-	-	-	-	-	-	-	5	-	-	-	-	-	-	-	-	-	-	5	-	-	-	-	-	-	-
Achillea millefolium	-	-	-	-	-	-	-	-	-	-	-	-	-	-	-	-	2	-	-	-	-	-	-	-	-	-	-	-	-	-	-	-	-	-	-	-	-	-	-	-	-	-	-	-	-	-	-	-	-	-	-	-	-	-	-	-	-	-	-	-	-	-	-	-	-	-	-	-	-	-	-	-	-	-	-	-	-	-	-	-	-	-	-	-
Achillea ptarmica	-	-	-	-	-	2	-	-	-	-	-	-	-	-	-	-	-	-	-	-	-	-	-	-	-	-	-	-	-	-	-	-	-	-	-	-	-	-	-	-	-	-	-	-	-	-	-	-	-	-	-	-	-	-	-	-	-	-	-	-	-	-	-	-	-	-	-	-	-	-	-	-	-	-	-	-	-	-	-	-	-	-	-	-
Acorus calamus	-	-	-	-	-	-	-	-	-	2	-	-	2	-	-	-	-	-	-	-	-	-	-	-	-	-	-	-	-	-	-	-	-	-	-	-	-	-	-	-	-	-	-	-	-	-	-	-	-	-	-	-	-	-	-	-	-	-	-	-	-	-	-	-	-	-	-	-	-	-	-	-	-	-	-	-	-	-	-	-	-	-	-	-
Aegopodium podagraria	-	-	-	-	-	-	-	-	-	-	-	-	-	-	-	-	-	-	-	-	-	-	-	-	2	6	-	6	-	-	-	-	-	-	-	-	1	-	2	-	-	-	-	-	-	-	-	-	-	-	-	-	-	-	-	-	-	-	-	-	-	-	-	1	4	-	2	-	-	-	-	-	-	-	-	-	2	-	-	-	-	-	-	-
Agrostis capillaris	-	-	-	-	-	-	-	-	-	-	-	-	-	-	-	-	-	-	-	7	-	-	-	-	-	-	-	-	-	-	-	-	-	-	-	-	-	-	-	-	-	-	-	-	-	-	-	-	-	-	-	-	-	-	-	-	-	-	-	-	-	-	-	-	-	-	-	-	-	-	-	-	-	-	-	-	-	-	-	-	-	-	-	-
Ajuga reptans	-	-	-	-	-	-	-	-	-	-	-	-	-	-	-	-	-	-	-	-	-	-	-	-	-	-	-	-	-	-	-	-	-	-	-	-	-	-	-	-	-	-	-	-	-	-	-	-	-	-	-	-	-	-	-	-	-	-	-	-	-	-	-	-	-	-	3	-	-	-	-	-	-	-	-	-	-	-	-	-	-	-	-	-
Alliaria petiolata	-	-	-	-	-	-	-	-	-	-	-	-	-	-	-	-	-	-	-	-	-	-	-	-	-	-	-	1	-	-	-	-	-	-	-	-	-	-	-	-	-	-	-	-	-	-	-	-	-	-	2	-	-	-	-	-	-	-	-	-	-	-	-	-	-	-	-	-	-	-	-	-	-	-	-	-	-	-	-	-	-	-	-	-
Allium oleraceum	-	-	-	-	-	-	-	-	-	-	-	-	-	-	-	-	-	1	-	-	-	-	-	-	-	-	-	-	-	-	-	-	-	-	-	-	-	-	-	-	-	-	-	-	-	-	-	-	-	-	-	-	-	-	-	-	-	-	-	-	-	-	-	-	-	-	-	-	-	-	-	-	-	-	-	-	-	-	-	-	-	-	-	-
Allium scorodoprasum	-	-	-	-	-	1	-	-	-	-	-	-	-	-	-	-	-	-	-	-	-	-	-	-	-	-	-	-	-	-	-	-	-	-	-	-	-	-	-	-	-	-	-	-	-	-	-	-	-	-	-	-	-	-	-	-	-	-	-	-	-	-	-	-	-	-	-	-	-	-	-	-	-	-	-	-	-	-	-	-	-	-	-	-
Alnus glutinosaA	-	-	-	-	-	-	-	-	-	-	-	-	-	-	-	-	-	-	-	-	-	-	-	-	-	-	-	-	-	-	-	-	-	-	-	-	-	-	-	-	-	-	-	-	-	-	-	-	-	-	-	-	-	-	-	-	-	-	-	-	-	7	2	8	-	-	9	6	-	7	2	7	-	-	-	-	-	-	-	-	-	3	-	-
Alnus glutinosaB	-	-	-	-	-	-	-	-	-	-	-	-	-	-	-	-	-	-	-	3	-	-	-	-	-	-	-	-	-	-	-	-	-	-	-	-	-	-	-	-	-	-	-	-	-	-	-	-	-	-	-	-	-	-	-	-	-	-	-	-	-	-	-	-	-	-	-	-	-	-	-	-	-	-	-	-	-	-	-	-	-	-	-	-
Alopecurus pratensis	-	-	3	2	-	3	-	-	-	-	2	-	-	2	-	3	1	-	-	-	-	-	-	-	-	-	-	-	-	-	-	-	-	-	-	-	-	-	-	-	-	-	-	-	-	-	-	-	2	-	2	-	-	-	-	2	-	-	-	-	-	-	-	2	-	2	-	-	2	-	2	-	-	-	-	6	2	-	6	2	-	-	-	-
Anemone nemorosa	-	-	-	-	-	-	-	-	-	-	-	-	-	-	-	-	-	1	-	-	-	-	-	-	-	-	-	-	-	-	-	-	-	-	-	-	-	-	-	-	-	-	-	-	-	-	-	-	-	-	-	-	-	-	-	-	-	-	-	-	-	2	-	2	-	-	-	-	-	-	-	-	-	-	-	-	-	-	-	-	-	-	-	-
Anthriscus sylvestris	-	-	-	-	-	-	-	-	-	-	-	-	-	-	-	-	-	-	-	-	-	-	-	-	-	-	-	2	-	-	-	-	-	-	-	-	-	-	-	-	-	-	-	-	-	-	-	-	-	-	-	-	-	-	-	-	-	-	2	-	-	-	-	-	2	-	-	-	-	-	-	-	-	-	-	-	-	-	-	-	-	-	-	-
Apios americana	-	-	-	-	-	-	-	-	-	-	-	-	-	-	-	-	-	-	-	-	-	-	-	-	-	-	-	-	-	-	-	-	-	-	-	-	-	-	-	-	-	-	-	-	-	-	-	-	-	-	-	-	-	-	-	-	-	-	-	-	-	-	-	-	-	-	-	-	-	-	-	-	-	-	-	-	-	-	-	-	2	-	-	-
Arrhenatherum elatius	-	-	-	-	-	-	-	2	-	-	-	-	-	-	-	-	-	-	-	-	-	-	-	-	-	-	-	-	-	-	-	-	-	-	-	-	-	-	-	-	-	2	-	2	-	-	-	-	-	-	-	-	-	-	-	-	-	-	-	-	-	-	-	-	-	-	-	-	3	-	-	-	-	-	-	2	-	-	2	-	-	-	-	-
Artemisia vulgaris	-	-	-	-	-	-	-	2	-	-	-	-	-	-	-	-	-	-	-	2	-	-	-	-	-	-	-	-	-	-	-	-	-	-	-	-	-	-	-	-	2	-	-	-	-	-	-	-	-	-	-	-	-	-	-	2	-	-	2	-	-	-	-	-	-	-	-	-	-	-	-	-	-	-	-	-	-	-	-	-	-	-	-	-
Aster lanceolatus	-	-	-	-	-	-	-	-	-	-	-	-	-	-	-	-	-	-	-	-	-	-	-	-	-	-	-	-	-	-	-	-	-	-	-	-	-	-	-	-	-	-	-	-	-	-	-	-	-	-	-	-	-	-	-	-	-	5	-	-	-	-	-	-	-	-	-	-	-	-	-	-	-	-	-	-	-	5	-	-	-	-	-	-
Bidens frondosa	-	-	-	-	-	-	-	-	-	-	-	-	1	-	-	-	-	-	-	-	-	-	-	2	-	-	-	-	-	-	-	-	-	-	-	-	-	-	-	-	-	-	-	-	-	-	-	-	-	-	1	-	-	-	-	-	-	-	-	-	-	-	-	-	-	-	-	-	-	-	-	-	-	-	-	-	-	-	-	-	-	-	-	-
Bilderdykia dumetorum	-	-	2	-	-	-	-	-	-	-	-	-	-	-	-	-	-	-	-	-	-	-	-	-	-	-	-	-	-	-	-	-	-	-	-	-	-	-	-	-	-	-	-	-	-	-	-	-	-	-	-	-	-	-	-	-	-	-	-	-	-	-	-	-	-	-	-	-	-	-	-	-	-	-	-	-	-	-	-	-	-	-	-	-
Bromus carinatus	-	-	-	-	-	-	-	-	-	-	-	-	-	-	-	-	-	-	-	-	-	-	-	-	-	-	3	-	-	-	-	-	-	-	-	-	-	-	-	-	-	-	-	-	-	-	-	-	-	-	-	-	-	-	-	-	-	-	-	-	-	-	-	-	-	-	-	-	-	-	-	-	-	-	-	-	-	-	-	-	-	-	-	-
Calamagrostis epigeios	-	-	-	-	-	-	-	-	-	-	-	-	-	-	-	2	2	-	-	-	-	-	-	-	-	-	-	-	-	-	-	-	-	-	-	-	-	-	-	-	-	-	-	-	-	-	-	-	-	-	-	-	-	-	-	-	-	-	-	-	-	-	-	-	-	-	-	-	-	-	-	-	-	-	-	-	-	-	-	-	-	-	-	-
Calystegia sepium	2	7	-	1	3	-	-	-	5	-	-	5	5	5	3	-	-	2	7	-	-	-	-	-	3	3	3	-	-	-	-	-	-	2	-	-	2	2	2	-	2	2	2	2	1	-	2	-	-	2	2	2	1	-	-	2	2	-	2	5	2	-	2	-	-	-	-	-	-	-	2	-	-	-	-	-	-	2	-	2	-	2	-	-
Carduus crispus	-	-	-	-	-	-	-	-	-	-	-	-	-	-	-	-	-	-	-	-	-	-	-	-	-	-	-	-	-	-	-	2	-	-	-	-	-	-	-	-	-	-	-	-	-	-	-	-	-	-	-	-	-	-	-	-	-	-	-	-	-	-	-	-	-	-	-	-	-	-	-	-	-	-	-	-	-	-	-	-	-	-	-	-
Carex acutiformis	-	-	-	-	-	-	-	-	-	2	-	-	-	-	-	-	-	-	-	-	-	-	-	-	-	-	-	-	-	-	-	-	-	-	-	-	-	-	-	-	-	-	-	-	-	-	-	-	-	-	-	-	-	-	-	-	-	-	-	-	-	-	-	-	-	-	-	-	-	-	-	-	-	-	-	-	-	-	-	-	3	2	-	3

Carex brizoides	- 6 - 7 - - - - - 7 - - - - - - - - - - 5 3 - 2
Carex buekii	8 8 9 9 8 8 9 8 9 9 9 9 9 9 9 9 9 9 8 8 7 8 7 9 8 7 9 7 8 8 9 8 8 8 9 9 9 9 8 9 9 9 9 8 9 9 9 8 8 9 9 9 8 9 8 8 9 9 9 9 9 8 8 7 9 9 9 9 9 7 7 9 9 9 9 8 9 8 8 9 7 8 8 8
Carex hirta	- 2 - 2 - - 2
Carex elata	- 1 - - - 2 1 2 - - - - - - - - - - - -
Carex praecox	- - - - - 3 - 2 -
Carex vesicaria	- 5 - - - - - - - - - - - - - - - -
Carex riparia	- 4 - - - - - - - - - -
Carpinus betulusA	- 2 -
Chaerophyllum bulbosum	- - - - - - - - - - - - - 2 -
Chelidonium majus	- 1 2 - 2 -
Cirsium arvense	- 3 - - - 7 2 3 2 - 2 - - 2 1 2 2 7 2 - - - - - - - - - - - - - - 2 2 - - - - 2 - - 1 - - - 2 - - - 1 - - 2 2 2 2 - 2 2 2 - - - - - - - 1 - - - - - 1 - - - - - - - - -
Cirsium oleraceum	- 2 -
Cirsium vulgare	- - - - - - - - - - - - - - - - - 2 -
Clematis vitalba	- 3 - 1 - - - - - - - - - - - - - - -
Cnidium dubium	- - - - - - - - - - - - - - - - 1 -
Convolvulus arvensis	- - - - - - - 3 - 2 - 1 - - - - - - - - - - - - - - -
Cornus sanguineaC	- - - - - - - - - - - - - - - - - - - 2 - 5 -
Crataegus laevigataC	- - - - - - - - - - - - - - - - - - - 3 -
Crataegus monogynaB	5 - 2 - - - - - 4 - - - - - - - - - - - - - -
Crataegus monogynaC	- - - - - - - - - - - - - - - - - 2 -
Cucubalus baccifer	- - - - - - - - 2 -
Dactylis glomerata	- 2 - 2 -
Deschampsia caespitosa	- - - - - 2 - 2 - 3 - - - - - - - - - - - - - - 2 - - - - - - -
Dryopteris carthusiana	- 1 - - - - - - - 1 - - - - - - - - - - - - - -
Elymus caninus	- 2 2 -
Elymus repens	- - 5 - - - - - - - - - - - - - - - 2 - - - - - - - - 1 - 2 - - - - - 2 - - - - - - - - -
Epipactis helleborine	- - - - 2 -
Equisetum arvense	2 - - - - 1 - 2 2 - - - - 2 - - 2 - - 2 - - - - - - - - - - - - - 1 - - - 2 - 2 - 1 - - - - - - - - - - - - - - -
Equisteum palustre	- 2 - - - - - - - - - - - - - - - - - - 1 - - - - - - - - - - - 2 - - - - - - - 1 - - - - -
Erigeron annuus	- 2 - - - - - - - -
Euonymus europaeusC	- 2 - - - - - - - - - 4 - - 1 - 3 - - - - - 4 - - - - - -
Eupatorium cannabinum	- 2 - - - - - - - - - 2 - 1 2 - - - -
Euphorbia esula	- 1 - - - - - - - - - - - - - - -
Festuca rubra	- - - - - - 2 - - - 1 -
Filipendula ulmaria	- - - - - 2 - - - - 2 - - 2 - 2 - 2 2 - - - - - - - - - - 5 1 - - - - - - 2 - - - - - - - 2 - - 1 - - 2 - - - - - - - - - - - - - - - - - - 1 - - - - - - - - - - 5 - 5
Filipendula vulgaris	- - - - - - 2 -
Fragaria vesca	- - - - - - 2 -
Fraxinus excelsiorC	- - 2 - 1 - - - - - - - - - - - - - - - - -
Galeobdolon luteum	- 2 -
Galeopsis bifida	- - 1 - 2 - 2 2 - - - - 2 -
Galeopsis pubescens	- 2 - - -
Galeopsis speciosa	- 2 - - 2 - - - - - - - - - 2 1 - 5 - - - - - 1 - - - - - - - - - - - - - -
Galeopsis tetrahit	- 2 - - - - - - - - - - - - - - - 2 -
Galium aparine	- - 3 - - - - 2 - - - - - - - - - - - - 2 - - - - 2 - - - - - 2 - 2 - - - - - - 2 2 2 1 2 - - 1 - - 9 - 2 - 2 2 2 2 2 - 2 2 3 3 3 - - - - - 2 2 1 - 1 - - 2 2 1 2 2 3 -
Galium boreale	- - - - - - 2 - - - - - - - - - 5 2 -
Galium elongatum	- 1 - - - - - - - - - - - - - - - -
Galium mollugo	2 - - - - - - 2 2 - 1 - 2 - - 2 - 1 - - - - - - - - - - 2 - - - - - - - -
Galium rivale	- 1 - - - 2 - 2 1 - 2 2 - 2 - 1 - - - - - - - - - - - - - - - - 2 - - - - - -

Galium rubioides	-	-	-	-	-	-	-	-	-	-	-	-	-	-	-	-	-	-	-	-	-	-	-	-	-	-	-	-	-	-	-	-	-	-	-	-	-	-	-	-	-	-	-	-	-	-	-	-	-	-	-	-	-	-	-	-	-	-	-	-	-	-	-	-	-	-	-	-	3	-	-	-	-	-	-	-	-	-	-	-	-	-	-	-
Galium verum	-	-	-	-	-	-	2	-	-	-	3	-	-	-	-	-	-	2	-	-	-	-	-	-	-	-	-	-	-	-	-	-	-	-	-	-	-	-	-	-	-	-	-	-	-	-	-	-	-	-	-	-	-	-	-	-	-	-	-	-	-	-	-	-	-	-	-	-	1	-	-	-	-	-	-	-	-	-	-	-	-	-	-	-
Geranium pratense	-	-	-	-	-	-	-	-	-	-	-	-	-	-	-	-	-	-	-	-	-	-	-	-	-	-	-	-	-	-	-	-	-	-	-	-	2	-	-	-	-	-	-	-	-	-	-	-	-	-	-	-	-	-	-	-	-	-	-	-	-	-	-	-	-	-	-	-	-	-	-	-	-	-	-	-	-	-	-	-	-	-	-	-
Geum urbanum	-	-	-	-	-	-	-	-	-	-	-	-	-	-	-	-	-	-	-	-	-	-	-	-	-	-	-	-	-	-	-	-	-	-	-	-	-	-	-	-	-	-	-	-	-	-	-	-	-	-	-	-	-	-	-	2	-	-	-	-	-	-	-	1	-	-	-	-	-	1	-	1	-	-	-	-	-	-	-	-	-	-	-	-
Helianthus tuberosus	-	-	-	-	-	-	-	-	-	-	-	-	-	-	-	-	-	-	-	-	-	-	-	-	-	1	-	-	-	-	-	-	-	-	-	-	-	-	-	-	-	-	-	-	-	-	-	-	-	-	-	-	-	-	-	-	-	-	-	-	-	-	-	-	-	-	-	-	-	-	-	-	-	-	-	-	-	-	-	-	-	-	-	-
**_Heracleum sphondylium_ subsp. _sphondylium_**	-	-	-	-	-	-	-	2	-	-	-	-	-	-	-	-	-	-	-	-	-	-	-	-	-	-	-	-	-	-	-	-	-	-	-	-	-	-	-	-	-	-	-	-	-	-	-	-	-	-	-	-	-	-	-	-	-	-	-	-	-	-	-	-	-	-	-	-	-	-	-	-	-	-	-	-	-	-	-	-	-	-	-	-
Hieracium umbellatum	-	-	-	-	-	-	-	-	-	-	-	-	-	-	-	-	-	-	-	2	-	-	-	-	-	-	-	-	-	-	-	-	-	-	-	-	-	-	-	-	-	-	-	-	-	-	-	-	-	-	-	-	-	-	-	-	-	-	-	-	-	-	-	-	-	-	-	-	-	-	-	-	-	-	-	-	-	-	-	-	-	-	-	-
Holcus lanatus	-	-	-	-	-	-	-	-	-	-	-	-	-	-	-	-	-	-	-	-	-	-	-	-	-	-	-	-	-	-	-	-	2	-	-	-	-	-	-	-	-	-	-	-	-	-	-	-	-	-	-	-	-	-	-	-	-	-	-	-	-	-	-	-	-	-	-	-	-	-	3	-	-	-	-	2	1	-	-	-	-	-	2	-
Humulus lupulus	-	-	-	-	-	-	-	-	2	-	-	-	-	2	-	-	-	3	3	-	-	-	-	-	-	-	-	-	-	-	-	-	-	-	-	-	2	-	6	-	-	-	2	-	-	-	-	-	-	1	-	5	-	-	-	2	-	-	-	-	2	-	-	2	2	-	2	-	-	2	-	1	-	-	-	-	2	-	-	-	-	2	3	-
Impatiens glandulifera	-	-	-	-	-	-	-	-	-	-	-	-	-	-	-	-	-	-	-	-	-	-	-	-	-	1	1	-	-	-	-	2	-	-	-	-	-	-	-	-	-	-	-	-	-	-	-	-	-	-	-	-	-	-	-	-	-	-	-	-	-	-	2	-	-	-	-	-	-	-	-	-	-	-	-	-	-	-	-	-	-	-	-	-
Impatiens noli-tangere	-	-	-	-	-	-	-	-	-	-	-	-	-	-	-	-	-	-	-	-	-	-	-	-	-	-	-	-	-	-	-	-	-	2	-	-	-	-	-	-	-	-	-	-	-	-	-	-	-	-	2	-	-	-	-	-	-	-	-	-	5	1	-	-	-	-	-	-	-	-	-	-	-	-	-	-	-	-	-	-	-	-	-	-
Impatiens parviflora	-	-	-	-	-	-	-	-	-	-	-	3	-	-	-	-	-	-	-	-	-	-	-	-	-	-	-	-	-	-	-	-	-	-	-	-	-	-	2	-	-	-	-	-	-	-	-	-	-	-	-	-	-	-	-	-	-	-	-	-	-	-	-	-	-	-	-	-	-	-	-	-	-	-	-	-	-	-	-	-	-	-	-	-
Inula britannica	-	-	-	-	-	2	-	-	-	-	-	-	-	-	-	-	-	-	-	-	-	-	-	-	-	-	-	-	-	-	-	-	-	-	-	-	-	-	-	-	-	-	-	-	-	-	-	-	-	-	-	-	-	-	-	-	-	-	-	-	-	-	-	-	-	-	-	-	-	-	-	-	-	-	-	-	-	-	-	-	-	-	-	-
Iris pseudoacorus	-	-	-	-	-	-	-	-	-	-	-	-	-	-	-	-	-	-	-	-	-	-	-	-	-	-	-	-	-	-	-	-	-	-	-	-	-	-	-	-	-	-	-	-	-	-	-	-	-	-	-	-	-	-	-	-	-	-	-	-	-	-	-	-	-	-	-	-	-	-	-	-	-	2	-	-	-	-	-	-	-	-	-	-
Juncus effusus	-	-	-	-	-	-	-	-	-	-	-	-	-	-	-	-	-	-	-	-	-	-	-	-	-	-	-	-	-	-	-	-	-	-	-	-	-	-	-	-	-	-	-	-	-	-	-	-	-	-	-	-	-	-	-	-	-	-	-	-	-	2	-	-	-	-	1	-	-	-	4	-	-	-	-	-	-	-	-	-	-	-	-	-
Knautia arvensis	-	-	-	-	-	-	-	-	-	-	-	-	-	-	-	-	-	-	-	-	-	-	-	-	-	-	-	-	-	-	-	-	-	-	-	-	-	-	-	-	-	-	-	-	-	-	-	-	-	-	-	-	-	-	-	-	-	-	-	-	-	-	-	-	-	-	-	-	1	-	-	-	-	-	-	-	-	-	-	-	-	-	-	-
Lamium maculatum	-	-	-	-	-	-	-	-	-	-	-	-	-	-	-	-	-	-	-	-	2	-	-	-	-	-	-	-	-	-	-	-	-	-	-	-	-	-	-	-	1	-	-	-	-	-	-	-	-	-	1	-	-	-	-	-	2	-	-	-	-	-	-	-	-	-	-	-	-	2	-	-	-	-	-	-	2	-	-	-	-	-	-	-
Lapsana communis	-	-	-	-	-	-	-	-	-	-	-	-	-	-	-	-	-	-	-	-	-	-	-	-	-	1	-	-	-	-	-	-	-	-	-	-	-	-	-	-	-	-	-	-	-	-	-	-	-	-	-	-	-	-	-	-	-	-	-	-	-	-	-	-	-	-	-	-	-	-	-	-	-	-	-	-	-	-	-	-	-	-	-	-
Lathyrus pratensis	-	-	-	-	-	-	-	-	2	-	2	-	-	-	-	1	-	2	-	-	-	-	-	-	-	-	-	-	-	-	-	-	-	-	-	-	-	-	-	-	-	-	1	-	-	-	-	-	-	-	-	-	-	-	2	-	-	-	-	-	-	-	-	-	-	-	-	-	-	-	1	-	-	-	-	2	-	-	2	-	-	-	-	-
Lathyrus tuberosus	-	-	-	-	-	-	-	-	-	-	-	-	-	-	-	-	-	-	-	-	-	-	-	-	-	-	-	-	-	-	-	-	-	-	-	-	-	-	-	-	-	-	-	-	-	-	-	-	-	-	-	-	-	-	-	-	-	-	-	-	-	-	-	-	-	-	-	-	1	-	-	-	-	-	-	-	-	-	-	-	-	-	-	-
Lonicera japonica	-	-	-	-	-	-	-	-	-	-	-	-	-	-	-	-	-	-	-	-	-	-	-	-	-	-	-	-	-	-	-	-	-	-	-	-	-	-	-	-	-	-	-	-	-	-	-	-	-	-	-	-	-	-	-	-	-	-	-	-	-	-	-	-	-	-	-	-	-	-	-	-	-	-	-	-	-	-	-	-	-	-	-	2
Lotus uliginosus	-	-	-	-	-	-	-	-	-	-	-	-	-	-	-	-	-	-	-	-	-	-	-	-	-	-	-	-	-	-	-	-	-	-	-	-	-	-	-	-	-	-	-	-	-	-	-	-	-	-	-	-	-	-	-	-	-	-	-	-	-	-	-	-	-	-	-	-	-	-	-	-	-	-	-	-	-	-	-	-	-	2	-	2
Lychnis flos-cuculi	-	-	-	-	-	-	-	-	-	-	-	-	-	-	-	-	-	-	-	-	-	-	-	-	-	-	-	-	-	-	-	-	-	-	-	-	-	-	-	-	-	-	-	-	-	-	-	-	-	-	-	-	-	-	-	-	-	-	-	-	-	1	-	-	-	1	-	-	-	-	2	-	-	-	2	-	1	-	-	-	-	-	-	-
Lycopus europaeus	-	-	-	-	-	-	-	-	-	-	-	-	1	-	-	-	-	-	-	-	-	-	-	-	-	-	-	-	-	-	-	-	-	-	-	-	-	-	-	-	-	-	-	-	-	-	-	-	-	-	-	-	-	2	-	-	-	-	-	-	-	-	-	-	-	-	-	-	-	-	-	-	-	-	-	-	-	-	-	-	-	-	-	-
Lysimachia nummularia	-	-	-	-	-	-	-	-	-	-	-	-	-	-	-	-	-	-	-	-	-	-	-	-	-	-	-	-	-	-	-	-	-	-	-	-	-	-	-	-	-	-	-	-	-	-	-	-	-	-	-	-	-	2	-	-	-	-	-	-	-	-	-	-	-	-	-	-	-	-	-	-	-	-	-	-	-	-	-	-	-	-	-	-
Lysimachia vulgaris	-	3	-	2	3	3	-	-	-	-	-	-	-	-	-	-	2	2	-	2	-	-	-	-	-	-	-	-	2	-	2	-	-	-	-	-	-	-	-	-	-	-	2	2	-	-	-	-	-	-	-	-	-	-	-	-	-	-	-	2	-	-	-	-	-	-	1	1	-	-	-	-	-	-	-	-	1	-	1	-	-	-	-	-
Lythrum salicaria	-	-	-	-	-	-	-	-	-	-	-	2	-	-	-	-	-	-	-	-	-	-	-	3	-	-	-	-	-	-	2	-	-	-	-	-	-	-	-	-	-	-	-	-	-	-	-	-	-	-	-	-	-	2	-	-	-	-	-	-	-	-	-	-	-	-	-	1	-	-	-	-	-	1	-	-	-	-	1	-	-	-	2	2
Mentha aquatica	-	-	-	-	-	-	-	-	-	-	-	-	-	-	-	-	-	-	-	-	-	-	-	-	-	-	-	-	-	-	-	-	-	-	-	-	-	-	-	-	-	-	-	-	-	-	-	-	-	-	-	-	-	-	-	-	-	-	-	-	-	-	-	-	-	-	1	-	-	-	2	-	-	-	-	-	-	-	-	-	-	-	-	-
Mentha longifolia	-	-	-	-	-	-	-	-	-	-	-	-	-	-	-	-	-	-	-	-	-	-	-	-	-	-	2	-	-	-	-	-	-	2	-	-	-	-	-	-	-	-	-	-	-	-	-	-	-	-	-	-	-	5	-	-	-	-	-	-	2	-	-	-	-	-	-	-	-	-	-	-	5	-	-	-	-	1	-	1	-	-	-	-
Myosotis palustris	-	-	-	-	-	-	-	-	-	-	-	-	-	-	-	-	-	-	-	-	-	-	-	2	-	-	-	-	-	-	-	-	-	-	-	-	-	-	-	-	-	-	-	-	-	-	-	-	-	-	-	-	-	-	-	-	-	-	-	-	1	-	-	-	-	-	-	-	-	-	2	-	-	-	-	-	1	-	-	-	-	-	-	-
Myososton aquaticum	-	-	-	-	-	-	-	-	-	-	-	-	-	-	-	-	-	-	-	-	-	-	-	-	-	-	-	-	-	-	-	-	-	-	-	-	-	-	-	-	2	-	-	-	-	-	-	-	-	-	-	-	-	-	-	-	-	-	-	-	-	2	-	-	-	-	-	-	-	-	-	-	-	-	2	1	2	-	-	1	-	-	-	-
Padus aviumA	-	-	-	-	-	-	-	-	-	-	-	-	-	-	-	-	-	-	-	-	-	-	-	-	-	-	-	-	-	-	-	-	-	-	-	-	-	-	-	-	-	-	-	-	-	-	-	-	-	-	-	-	-	-	-	-	-	-	-	-	-	-	-	-	3	-	-	4	-	-	-	-	-	-	-	-	8	7	6	-	-	-	-	-
Padus aviumB	-	-	-	-	-	-	-	-	-	-	-	-	-	-	-	-	-	-	-	-	-	-	-	-	-	-	-	-	-	-	-	-	-	-	-	-	-	-	-	-	-	-	-	-	-	-	-	-	-	-	-	-	-	-	-	-	-	-	-	-	-	1	-	-	-	-	-	-	-	-	-	-	-	-	-	-	-	-	-	-	-	1	-	-
Petasites hybridus	-	-	-	-	-	-	-	-	-	-	-	-	-	-	-	-	-	-	-	-	-	-	-	-	-	-	-	-	-	-	-	-	-	-	-	-	-	-	-	-	-	-	-	-	-	-	-	-	-	-	-	-	-	-	-	-	-	-	-	-	1	-	-	-	-	-	4	-	-	-	-	-	-	-	-	-	4	-	-	-	-	-	-	-
Peucedanum palustre	-	-	-	-	-	-	-	-	-	-	-	-	-	-	-	-	-	-	-	-	-	-	-	-	-	-	-	-	-	-	-	-	-	-	-	-	-	-	-	-	-	-	-	-	-	-	-	-	-	-	-	-	-	-	-	-	-	-	-	-	-	-	-	-	-	-	-	-	-	1	-	-	-	-	-	-	-	-	-	-	-	-	-	-
Phalaris arundinacea	3	-	-	3	7	-	-	-	-	-	3	2	2	3	3	-	-	-	2	-	-	-	-	2	2	2	5	-	6	5	5	-	-	-	-	-	-	-	-	-	-	-	2	6	-	1	1	-	2	-	1	2	2	-	-	-	-	-	2	-	-	-	-	-	-	-	-	-	-	-	-	4	-	5	-	-	-	3	-	-	-	-	-	2
Phragmites australis	1	5	-	-	-	-	-	-	-	-	-	-	-	-	-	-	-	2	2	-	3	-	-	-	-	-	-	-	-	-	-	-	-	-	-	-	-	-	-	-	-	-	-	-	-	-	-	-	-	-	-	-	-	-	-	-	-	-	-	-	-	-	-	-	-	-	-	-	-	-	-	-	-	-	-	5	-	-	-	-	-	-	-	-
Poa palustris	-	-	-	-	-	-	-	-	-	-	-	-	-	-	-	-	2	-	-	-	-	-	-	-	-	-	-	-	-	-	-	-	-	-	-	-	-	-	-	-	-	-	-	-	-	-	-	-	-	-	-	-	-	-	-	-	-	-	-	-	-	-	-	-	-	-	-	-	-	-	-	-	-	-	-	-	-	-	-	-	-	-	-	-
Poa pratensis	-	-	-	-	-	3	-	-	-	-	-	-	-	-	-	-	-	-	-	2	-	-	-	-	-	-	-	-	-	-	-	-	-	-	-	-	-	-	-	-	-	-	-	-	-	-	-	-	-	-	-	-	-	-	-	-	-	-	-	-	-	-	-	-	-	2	-	-	-	-	-	-	-	-	-	2	-	-	2	-	-	-	-	-
Poa trivialis	-	-	-	-	-	-	-	-	-	-	-	-	-	-	-	-	-	-	-	-	-	-	-	-	-	-	-	-	-	-	-	-	-	-	-	-	-	-	-	-	2	-	-	-	-	-	-	-	-	-	2	-	2	2	-	2	2	-	-	-	2	-	-	3	-	1	-	-	-	3	5	-	-	-	1	-	1	3	-	1	-	-	-	-
**_Polygonum amphibium_ f. _terrestre_**	-	2	-	-	-	-	-	2	-	-	-	-	-	-	-	-	-	-	2	-	-	-	-	-	-	-	-	-	-	-	-	-	-	-	-	-	-	-	-	-	-	-	-	-	-	-	-	-	-	-	-	-	-	-	-	-	-	-	-	-	-	-	-	-	-	-	-	-	-	-	-	-	-	-	-	-	-	-	-	-	-	-	-	-
Polygonum bistorta	-	-	-	-	-	-	-	-	-	1	-	-	-	-	-	-	-	-	-	-	-	-	-	-	-	-	-	-	-	-	-	-	-	-	-	-	-	-	1	-	-	-	-	-	-	-	-	-	-	-	-	-	-	-	-	-	-	-	-	-	-	-	-	-	-	-	-	-	-	-	-	-	-	-	-	-	-	-	-	-	-	-	-	-
Polygonum hydropiper	-	-	-	-	-	-	-	-	-	-	-	-	-	-	-	-	-	-	-	-	-	-	-	2	-	-	-	-	-	-	-	-	-	-	-	-	-	-	-	-	-	-	-	-	-	-	-	-	-	-	2	-	-	-	-	-	-	-	-	-	-	2	-	-	-	-	-	-	-	2	-	-	-	1	-	-	-	1	-	-	-	-	-	-
Populus tremulaA	-	-	-	-	-	-	-	-	-	-	-	-	-	-	-	-	-	-	-	-	-	-	-	-	-	-	-	-	-	-	-	-	-	-	-	-	-	-	-	-	-	-	-	-	-	-	-	-	-	-	-	-	-	-	-	-	-	-	-	-	-	3	-	-	-	-	-	-	-	-	-	-	-	-	-	-	-	-	-	-	-	-	-	-
Potentilla reptans	-	-	-	-	-	-	-	-	-	2	-	-	-	-	-	-	1	-	-	-	-	-	-	-	-	-	-	-	-	-	-	-	-	-	-	-	-	-	-	-	-	-	-	-	-	-	-	-	-	-	-	-	-	-	-	-	-	-	-	-	-	-	-	-	-	-	-	-	-	-	-	-	-	-	-	-	-	-	-	-	-	-	-	-
Prunus spinosaB	-	-	-	-	-	-	1	-	-	-	-	-	-	-	-	-	1	-	-	-	-	-	-	-	-	-	-	-	-	-	-	-	-	-	-	-	-	-	-	-	-	-	-	-	-	-	-	-	-	-	-	-	-	-	-	-	-	-	-	-	-	-	-	-	-	-	-	-	-	-	-	-	-	-	-	-	-	-	-	-	-	-	-	-

Prunus spinosaC	-	-	-	-	-	-	-	-	-	-	-	-	-	-	-	-	-	-	-	-	-	-	-	-	-	-	-	-	-	-	-	-	-	-	-	-	-	-	-	-	1	-	-	-	-	-	-	-	-	-	-	-	-	-	-	-	-	-	-	-	-	-	-	-	-	-	-	-	-	-	-	-	-	-	-	-	-	-	-	-	-	-	-	-
Pyrus communisC	-	-	-	-	-	1	-	-	-	-	-	-	-	-	-	-	-	-	-	-	-	-	-	-	-	-	-	-	-	-	-	-	-	-	-	-	-	-	-	-	-	-	-	-	-	-	-	-	-	-	-	-	-	-	-	-	-	-	-	-	-	-	-	-	-	-	-	-	-	-	-	-	-	-	-	-	-	-	-	-	-	-	-	-
Quercus roburA	-	-	-	-	-	-	-	-	-	-	-	-	-	-	-	-	-	-	-	7	-	-	-	-	-	-	-	-	-	-	-	-	-	-	-	-	-	-	-	-	-	-	-	-	-	-	-	-	-	-	-	-	-	-	-	-	-	-	-	-	-	-	-	-	-	-	-	-	-	-	-	-	-	-	-	-	-	-	-	-	-	-	3	-
Quercus roburC	-	-	-	-	-	-	-	-	-	-	-	-	-	-	-	-	-	-	-	2	-	-	-	-	-	-	-	-	-	-	-	-	-	-	-	-	-	-	-	-	-	-	-	-	-	-	-	-	-	-	-	-	-	-	-	-	-	-	-	-	-	-	-	-	-	-	-	-	-	-	-	-	-	-	-	-	-	-	-	-	-	-	-	-
Ranunculus acris	-	-	-	-	-	-	-	-	-	-	-	-	-	-	-	-	-	-	-	-	-	-	-	-	-	-	-	-	-	-	-	-	-	-	-	-	-	-	-	-	-	-	-	-	-	-	-	-	-	-	-	-	-	-	-	-	-	-	-	-	-	-	-	-	-	-	-	-	-	-	1	-	-	-	-	1	-	1	1	-	-	-	-	-
Ranunculus ficaria	-	-	-	-	-	-	-	-	-	-	-	-	-	-	-	-	-	-	-	-	-	-	-	-	-	-	-	-	-	-	-	-	-	-	-	-	-	-	-	-	-	-	-	-	-	1	-	-	-	-	-	-	-	-	-	-	-	-	-	-	-	-	-	-	-	-	-	-	-	-	-	-	-	-	-	-	-	-	-	-	-	-	-	-
Ranunculus repens	-	-	-	-	-	-	-	-	-	2	-	-	-	-	-	-	-	-	-	-	-	-	-	-	-	-	-	-	-	-	-	-	-	-	-	-	-	-	-	-	-	-	-	-	-	-	-	-	-	-	-	-	-	-	-	-	-	-	2	-	-	-	-	-	-	-	-	-	-	-	-	-	-	-	-	-	-	-	-	-	-	-	-	-
Reynoutria japonica	-	-	-	-	-	-	-	-	-	-	-	-	-	-	-	-	-	-	-	-	-	-	-	-	-	-	2	-	-	-	-	-	-	-	-	-	-	-	-	-	-	-	-	-	-	-	-	-	-	-	-	-	-	-	-	-	-	-	-	-	-	-	-	-	-	-	-	-	-	-	-	-	-	-	-	-	-	-	-	-	-	-	-	-
Rhamnus catharticusC	-	-	-	-	-	2	1	-	-	-	-	-	-	-	-	-	-	-	-	-	-	-	-	-	-	-	-	-	-	-	-	-	-	-	-	-	-	-	-	-	-	-	-	-	-	-	-	-	-	-	-	-	-	-	-	-	-	-	-	-	-	-	-	-	-	-	-	-	-	-	-	-	-	-	-	-	-	-	-	-	-	-	-	-
Rosa caninaB	3	-	-	-	-	-	-	-	-	-	-	-	-	-	-	-	-	-	-	-	-	-	-	-	-	-	-	-	-	-	-	-	-	-	-	-	-	-	-	-	-	-	-	-	-	-	-	-	-	-	-	-	-	-	-	-	-	-	-	-	-	-	-	-	-	-	-	-	-	-	-	-	-	-	-	-	-	-	-	-	-	-	-	-
Rosa caninaC	-	-	-	-	-	-	-	-	-	-	-	-	-	-	-	-	1	-	-	-	-	-	-	-	-	-	-	-	-	-	-	-	-	-	-	-	-	-	-	-	-	-	-	-	-	-	-	-	-	-	-	-	-	-	-	-	-	-	-	-	-	-	-	-	-	-	-	-	-	-	-	-	-	-	-	-	-	-	-	-	-	-	-	-
Rosa gallicaC	-	-	-	-	-	-	-	-	-	-	-	-	-	-	-	-	-	-	-	-	-	-	-	-	-	-	-	-	-	-	-	-	-	-	-	-	-	-	-	-	-	-	-	-	-	-	-	-	-	-	-	-	-	-	-	-	-	-	-	-	-	-	-	-	-	-	-	-	-	-	-	-	-	-	-	-	-	-	-	-	-	-	-	-
Rubus caesiusC	7	3	-	2	2	-	-	7	3	-	-	2	-	5	3	-	2	2	3	3	2	-	-	-	2	2	3	-	-	-	-	-	-	-	-	-	2	-	2	-	-	-	2	-	2	-	-	-	-	-	2	-	-	-	-	1	-	-	-	-	-	2	-	2	5	3	-	-	-	-	-	-	-	-	-	-	-	-	-	-	5	3	-	2
Rubus idaeusC	-	-	-	-	-	-	-	-	-	-	-	-	-	-	-	-	-	-	-	-	2	-	-	2	-	-	-	-	-	-	-	2	-	-	-	-	-	-	-	-	-	-	2	-	-	-	-	-	-	-	-	-	-	-	-	2	-	-	-	-	-	-	-	-	-	-	-	-	-	-	-	-	-	-	-	-	-	4	-	-	-	-	-	-
Rubus fruticosus agg.***C***	-	-	-	-	-	-	-	-	-	-	-	-	-	-	-	-	-	-	-	-	-	-	-	-	-	-	-	-	-	-	-	-	-	-	-	-	-	-	-	-	-	-	-	-	-	-	-	-	-	5	-	-	-	-	-	-	-	-	-	-	-	-	-	-	-	-	2	-	-	2	-	-	-	-	-	-	-	-	-	-	-	-	-	-
Rubus ulmifoliusC	-	-	-	-	-	-	-	-	-	-	-	-	-	-	-	-	-	-	-	-	-	-	-	-	-	-	-	-	-	-	-	-	-	-	-	-	-	-	-	-	-	-	-	-	-	-	-	-	-	-	-	-	-	-	-	-	-	-	-	-	-	-	-	-	-	-	-	-	-	-	-	-	-	-	-	-	-	-	-	-	-	-	5	-
Rudbeckia laciniata	-	-	-	-	-	-	-	-	-	-	-	-	-	-	-	-	-	-	-	-	-	-	-	-	-	-	-	-	-	-	-	-	-	-	-	-	-	-	-	-	-	-	-	-	-	-	-	-	-	-	-	-	-	-	-	-	-	-	-	-	-	-	-	-	-	-	4	-	-	-	-	-	-	-	-	-	-	-	-	-	-	-	-	-
Rumex acetosa	-	-	-	-	-	-	-	-	-	-	2	-	-	-	-	-	-	-	-	2	-	-	-	-	-	-	-	-	-	-	-	-	-	-	-	-	-	-	2	-	-	-	-	-	-	-	-	-	-	-	-	-	-	-	-	-	-	-	-	-	-	-	-	-	-	-	-	-	-	-	1	-	-	-	-	2	-	-	-	-	-	-	-	-
Rumex thyrsiflorus	-	-	-	-	-	-	-	-	-	-	-	-	-	-	-	-	-	-	-	-	-	-	-	-	-	-	-	-	-	-	-	-	-	-	-	-	-	-	-	-	-	-	-	-	-	-	-	-	-	-	-	-	-	-	-	-	-	-	-	-	-	-	-	-	-	-	-	-	1	-	-	-	-	-	1	-	-	-	1	-	-	-	-	-
Salix fragilis A	-	-	-	-	-	-	-	-	-	-	-	-	-	-	-	-	-	-	-	-	-	-	-	-	-	-	-	-	-	-	-	-	-	-	-	-	-	-	-	-	-	-	-	-	-	-	-	-	-	-	-	-	-	-	-	-	-	-	-	-	-	-	-	-	3	-	-	-	-	-	-	6	-	-	-	-	-	-	-	-	-	-	-	-
Salix alba A	-	-	-	-	-	-	-	-	-	-	-	-	-	-	-	-	-	-	-	-	-	-	-	-	-	-	-	-	-	-	-	-	-	-	-	-	-	-	-	-	-	-	-	-	-	-	-	-	-	-	-	-	-	-	-	-	-	-	-	-	-	-	5	-	-	6	-	-	-	-	-	-	-	3	-	-	-	-	-	6	3	-	-	-
Sambucus nigraC	-	-	-	-	-	-	-	-	-	-	-	-	-	-	-	-	-	-	-	-	-	-	-	-	-	-	-	-	-	-	-	-	-	-	-	-	-	-	-	-	-	-	-	-	-	-	-	-	-	-	-	-	-	-	-	-	-	-	-	-	-	1	-	-	-	-	3	-	-	-	-	-	-	-	-	-	-	-	-	-	-	-	-	-
Sanguisorba officinalis	-	3	-	2	-	2	2	-	-	-	-	-	-	-	-	2	-	-	2	-	-	-	-	-	-	-	-	-	-	-	-	-	-	-	-	-	-	-	2	-	-	-	-	-	-	-	-	-	-	-	-	-	-	-	-	-	-	-	-	-	-	-	-	-	-	-	-	-	-	-	-	-	-	-	-	-	-	-	-	-	-	-	-	-
Saponaria officinalis	-	-	-	-	-	-	-	-	-	-	-	-	-	-	-	-	-	-	-	-	-	-	-	-	-	-	-	-	-	-	-	-	-	-	-	-	-	-	-	-	-	-	-	-	-	-	-	-	-	-	-	-	-	-	-	-	-	-	2	-	-	-	-	-	-	-	-	-	-	-	-	-	-	-	-	-	-	-	-	-	-	-	-	-
Scirpus sylvaticus	-	-	-	-	-	-	-	-	-	-	-	-	-	-	-	-	-	-	-	-	-	-	-	-	-	-	-	-	-	-	-	-	-	-	-	-	-	-	-	-	-	-	-	-	-	-	-	-	-	-	-	-	-	-	-	-	-	-	-	-	-	-	-	-	-	-	-	2	-	-	5	-	-	-	-	-	-	-	-	-	5	2	-	3
Scrophularia nodosa	-	-	-	-	-	5	-	-	-	-	-	-	-	-	-	-	-	-	-	-	-	-	-	-	-	-	-	-	-	-	-	-	-	-	-	-	-	-	-	-	-	2	-	-	-	-	-	-	-	-	-	-	-	-	-	-	-	-	-	-	-	-	-	1	-	-	-	-	-	-	-	-	-	-	-	-	-	-	-	-	-	-	-	-
Scrophularia umbrosa	-	-	-	-	-	-	-	-	-	-	-	-	-	-	-	-	-	-	-	-	-	-	-	-	-	-	-	-	-	-	-	-	-	-	-	-	-	-	-	-	-	-	-	-	-	-	-	-	-	-	-	-	-	-	-	-	-	-	-	-	-	-	-	-	-	-	-	-	-	-	1	-	-	-	-	-	-	-	-	-	-	-	-	-
Scutellaria galericulata	-	-	-	-	-	-	-	-	-	-	-	-	-	-	-	-	-	-	-	-	-	-	-	-	-	-	-	-	-	-	-	-	-	-	-	-	-	-	-	-	-	-	-	-	-	-	-	-	-	-	-	-	-	-	-	-	-	-	-	-	-	-	-	-	-	-	-	-	-	-	-	-	1	-	1	1	-	-	-	-	-	-	-	-
Scutellaria hastifolia	-	-	-	-	-	3	-	-	-	-	-	-	-	-	-	-	-	-	-	-	-	-	-	-	-	-	-	-	-	-	-	-	-	-	-	-	-	-	-	-	-	-	-	-	-	-	-	-	-	-	-	-	-	-	-	-	-	-	-	-	-	-	-	-	-	-	-	-	-	-	-	-	-	-	-	-	-	-	-	-	-	-	-	-
Selinum carvifolia	-	-	-	-	-	3	-	-	-	-	-	-	-	-	-	-	-	-	-	-	-	-	-	-	-	-	-	-	-	-	-	-	-	-	-	-	-	-	-	-	-	-	-	-	-	-	-	-	-	-	-	-	-	-	-	-	-	-	-	-	-	-	-	-	-	-	-	-	-	-	-	-	-	-	-	-	-	-	-	-	-	-	-	-
Senecio ovatus	-	-	-	-	-	-	-	-	-	-	-	-	-	-	-	-	-	-	-	-	-	-	-	-	-	-	-	-	-	-	-	-	-	2	-	-	-	-	-	-	-	-	-	-	-	-	-	-	-	-	-	-	-	-	-	-	-	-	-	-	-	-	-	-	-	-	-	-	-	-	-	-	-	-	-	-	-	-	-	-	-	-	-	-
Serratula tinctoria	-	-	-	1	-	-	-	-	-	-	-	-	-	-	-	2	-	-	-	-	-	-	-	-	-	-	-	-	-	-	-	-	-	-	-	-	-	-	-	-	-	-	-	-	-	-	-	-	-	-	-	-	-	-	-	-	-	-	-	-	-	-	-	-	-	-	-	-	-	-	-	-	-	-	-	-	-	-	-	-	-	-	-	-
Silene alba	-	-	-	-	-	-	-	-	-	-	-	-	-	-	-	-	-	-	-	-	-	-	-	-	-	-	-	-	-	-	-	-	-	-	-	-	-	-	-	-	2	-	1	-	-	-	-	-	-	-	-	-	-	-	-	-	-	-	-	-	-	-	-	-	-	-	-	-	-	-	-	-	-	-	-	-	-	-	-	-	-	-	-	-
Sium latifolium	-	-	-	-	-	-	-	-	-	-	-	-	-	-	-	-	-	-	-	-	-	-	-	-	-	-	-	-	-	-	-	-	-	-	-	-	-	-	-	-	-	-	-	-	-	-	-	-	-	-	-	-	-	-	-	-	-	-	-	-	-	-	-	-	-	-	-	-	-	-	1	-	-	-	-	-	-	-	-	-	-	-	-	-
Solanum dulcamara	-	-	-	-	-	-	-	-	-	-	-	-	-	-	-	-	-	-	-	-	-	-	-	-	-	-	-	-	-	-	-	-	-	-	-	-	-	-	-	-	-	-	-	-	-	-	-	-	-	-	-	-	-	-	-	-	-	-	-	-	-	-	-	-	-	-	-	-	-	-	-	-	1	-	-	1	-	1	-	-	-	-	-	-
Solidago gigantea	-	2	-	-	-	2	-	2	-	-	-	-	-	2	3	-	1	-	-	-	-	-	-	-	-	-	-	-	-	-	-	-	-	-	-	-	-	-	-	-	-	-	2	-	-	-	-	-	-	-	-	-	-	-	-	-	-	-	-	-	-	2	5	2	6	-	-	5	-	-	2	2	-	-	3	-	1	-	-	5	-	-	-	-
Sonchus arvensis	-	-	-	-	-	-	-	-	-	-	-	-	-	-	-	-	-	-	-	-	-	-	-	-	-	-	-	-	-	-	-	-	-	-	-	-	-	-	-	-	-	-	-	-	-	-	-	-	-	-	-	-	-	-	-	-	-	-	-	-	-	-	-	-	-	-	-	-	-	-	-	-	-	-	1	-	-	-	-	-	-	-	-	-
Sonchus palustris	-	-	-	-	-	-	-	-	-	-	-	-	-	-	-	-	-	-	-	-	-	-	-	-	-	-	-	-	-	-	-	-	-	-	-	-	-	-	-	-	-	-	-	-	-	-	-	-	-	-	-	-	-	-	-	-	-	-	-	-	-	-	-	-	-	-	-	-	-	-	-	-	2	-	-	-	-	-	-	-	-	-	-	-
Sparganium erectum	-	-	-	-	-	-	-	-	-	-	-	-	-	-	-	-	-	-	-	-	-	-	-	-	-	-	-	-	-	-	-	-	-	-	-	-	-	-	-	-	-	-	-	-	-	-	-	-	-	-	-	-	-	-	-	-	-	-	-	-	-	-	-	-	-	-	-	-	-	-	-	-	-	-	-	-	-	-	-	-	3	-	-	3
Stachys palustris	-	-	-	-	-	-	-	-	-	-	-	-	-	-	-	-	-	-	-	-	-	-	-	-	-	-	-	-	-	-	-	-	-	-	-	-	-	-	-	-	-	-	-	-	-	-	-	-	-	-	-	-	-	-	-	-	-	-	-	-	-	-	-	-	-	-	-	-	-	-	-	-	-	-	-	-	-	-	-	1	-	-	-	-
Stachys sylvatica	-	-	-	-	-	-	-	-	-	-	-	-	-	-	-	-	-	-	-	-	2	-	-	-	-	-	-	-	-	-	-	-	-	-	-	-	-	-	-	-	-	-	-	-	-	-	-	-	-	-	-	-	-	-	-	-	-	-	-	-	-	-	-	-	-	-	1	-	-	-	-	-	-	-	-	-	-	-	-	-	-	-	-	-
Stellaria graminea	-	-	-	-	-	-	-	-	-	-	-	-	-	-	-	-	-	-	-	-	-	-	-	-	-	-	-	-	-	-	-	-	-	-	-	-	-	-	-	-	-	-	-	-	-	-	-	-	-	-	-	-	-	-	-	-	-	-	-	-	-	-	-	-	-	1	-	-	-	-	-	-	-	-	-	-	1	-	2	-	-	-	-	-
Stellaria media	-	-	-	-	-	-	-	-	-	-	-	-	-	-	-	-	-	-	-	-	-	-	-	-	-	-	-	-	-	-	-	-	-	-	-	-	-	-	-	-	-	-	-	-	-	-	-	-	-	-	-	-	-	-	-	-	-	-	-	-	-	-	-	1	-	-	-	-	-	-	-	-	-	-	1	-	-	-	-	-	-	-	-	-
Stellaria neglecta	-	-	-	-	-	-	-	-	-	-	-	-	-	-	-	-	-	-	-	-	-	-	-	-	-	-	-	-	-	-	-	-	-	-	-	-	-	-	-	-	-	-	-	-	-	-	-	-	-	-	-	-	-	-	-	-	-	-	-	-	-	-	-	-	-	-	-	-	-	2	-	-	-	-	-	-	-	-	-	-	-	-	-	-
Stellaria nemorum	-	-	-	-	-	-	-	-	-	-	-	-	-	-	-	-	-	-	-	-	-	-	-	-	-	6	-	-	-	-	-	-	-	-	-	-	-	-	-	-	-	-	-	-	-	-	-	-	-	-	-	-	-	-	-	-	-	-	-	-	-	-	-	-	-	-	-	-	-	-	-	-	-	-	-	-	-	-	-	-	-	-	-	-
Symphytum officinale	-	-	-	-	2	3	-	-	-	-	-	2	3	-	2	-	-	3	-	-	-	-	-	-	-	-	-	-	2	2	-	-	-	-	-	-	-	-	-	-	-	-	-	-	-	-	-	-	-	-	-	-	-	2	-	2	-	-	-	-	2	-	-	-	-	-	-	-	-	-	1	-	-	3	-	-	2	-	-	1	-	-	-	-
Tanacetum vulgare	-	-	-	-	-	-	-	-	2	-	-	-	-	-	-	-	3	-	-	-	-	-	-	-	-	-	-	-	-	-	-	-	-	-	-	-	-	-	-	-	-	-	-	-	-	-	-	-	-	-	-	-	-	-	-	-	-	-	-	-	-	1	-	-	-	-	-	-	-	-	-	-	-	-	1	3	-	-	3	-	-	-	-	-
Thalictrum lucidum	-	-	-	-	-	-	-	-	-	-	-	-	-	-	-	2	-	-	-	-	-	-	-	-	-	-	-	-	-	-	-	-	-	-	-	-	-	-	-	-	-	-	-	-	-	-	-	-	-	-	-	-	-	-	-	-	-	-	-	-	-	-	-	-	-	-	-	-	-	-	-	-	-	-	-	-	-	-	-	-	-	-	-	-

Tragopogon orientalis	- 1 - - - - - - - - - - - - - - -
Urtica dioica	**3 - 5 - 2 - 2 2 1 - - 2 2 - 2 - - 2 8 - 6 - - 2 - - 3 - 2 5 1 6 - 6 2 - 2 - - - 1 - 1 - - - 6 1 5 - 5 2 6 - 5 5 2 2 5 - 1 2 6 5 2 4 - - - 3 3 3 - 2 - - 2 - 3 2 3 5 2 2**
Veronica beccabunga	- 1 -
Veronica longifolia	**, - - - - - - - - - - - - - - 2 - 2 -**
Viburnum opulusC	**- - - - - - - - - - - - - - - - - - - 2 - 2 - - - - - - - 3 - - - - - - - 5 - - - - - -**
Vicia cracca	**3 2 - 2 3 2 - - - - - - 3 - 2 2 2 2 - - - - - 2 - - - - 2 2 - - - - - - 2 - - - - - - - - - - 2 - - 2 1 - - - - - - 2 - - - - - - - - - 1 - - - 1 - - - - - 1 - - - - -**
Vicia sepium	- 6 - - - - - - - - - - 6 - - - - - - -
Vicia tetrasperma	- - - - - - - - - - - - - - - - 3 -
Vincetoxicum hirundinaria	- - - - - 3 -
Viola canina	- - - - - - - - - 3 - - 3 -
