# Supplementary material for: Ecology, threats and conservation status of Carex buekii (Cyperaceae) in Central Europe
Source: Sci Rep. 2019 Aug 1;9:11162. doi: 10.1038/s41598-019-47563-0 (PMC6671956; doi:10.1038/s41598-019-47563-0)
Supplement: Supplementary file 3 — Differences in habitat conditions at Carex buekii sites sampled [file 41598_2019_47563_MOESM3_ESM.pdf]

Supplementary Table S3

Ecology, threats and conservation status of *Carex buekii* (Cyperaceae) in Central Europe

Helena Więcław[1], Kateřina Šumberová[2], Beata Bosiacka[1], Richard Hrivnák[3], Zygmunt Dajdok[4], Attila Mesterházy[5], Chiara Minuzzo[6], Edoardo Martinetto[7], Jacob Koopman[8]

[1]Institute of Marine & Environmental Sciences, University of Szczecin, Wąska 13, PL-71-415 Szczecin, Poland;
[2] The Czech Academy of Sciences, Institute of Botany, Department of Vegetation Ecology, Lidická 25/27, CZ-602 00 Brno, Czech Republic;
[3]Institute of Botany, Plant Science and Biodiversity Center, Slovak Academy of Sciences, Dúbravská cesta 9, SK-845 23 Bratislava, Slovakia;
[4]Institute of Environmental Biology, University of Wrocław, Kanonia 6/8, PL-50-328 Wrocław, Poland;
[5]Directorate of Hortobágy National Park, H-4024 Sumen utca 2, Debrecen, Hungary;
[6]Regione Serramonte 10, I-10010 Andrate, Italy,
[7]Earth Sciences Department, University of Turin, Via Valperga Caluso 35, I-10125 Turin, Italy,
[8]ul. Kochanowskiego 27, PL-73-200 Choszczno, Poland

e-mail: helena.wieclaw@usz.edu.pl

Results of Kruskal–Wallis test and *post hoc* Dunn's multiple comparisons test, showing significance of differences in habitat conditions at *Carex buekii* sites sampled. P – Poland, CR - Czech Republic, S – Slovakia, H – Hungary, I – Italy; C/N – ratio between organic carbon and nitrogen; org. mat. – organic matter content; pH – soil pH; Ca – calcium; Mg – magnesium; P – phosphorus; K – potassium; ECe – electrolytic conductivity; H - values critical, p - statistical significance. Significance level p ≤ 0.05 marked in bold.

Environmental variables	Kruskal-Wallis test		Dunn's multiple comparisons test									
			P-CR	P-S	P-H	P-I	CR-S	CR-H	CR-I	S-H	S-I	H-I
	H	*p*	*p*	*p*	*p*	*p*	*p*	*p*	*p*	*p*	*p*	*p*
elevation [a.s.l.]	45.22	**0.0000**	**0.0000**	**0.0000**	**0.0005**	**0.0121**	1.0000	1.0000	1.0000	1.0000	1.0000	1.0000
precipitation [mm]	29.60	**0.0000**	1.0000	**0.0417**	**0.0042**	**0.0004**	0.2812	**0.0406**	**0.0022**	1.0000	0.1409	0.4462
temperature [°C]	26.73	**0.0000**	0.1402	1.0000	**0.0062**	**0.0004**	1.0000	1.0000	0.0697	0.1179	**0.0034**	0.3518
pH	20.35	**0.0004**	1.0000	**0.0002**	0.3334	1.0000	**0.0212**	1.0000	1.0000	0.4324	0.2629	1.0000
K [mg/kg]	19.18	**0.0007**	1.0000	1.0000	**0.0131**	**0.0359**	1.0000	0.1498	0.1409	**0.0239**	0.0514	1.0000
silt	18.05	**0.0012**	0.1212	1.0000	1.0000	1.0000	**0.0047**	**0.0013**	1.0000	1.0000	1.0000	1.0000
clay	17.88	**0.0013**	**0.0434**	**0.0095**	1.0000	0.0788	1.0000	0.5099	1.0000	0.1703	1.0000	0.3215
C/N	17.13	**0.0018**	1.0000	0.7598	**0.0020**	0.1499	1.0000	0.0838	0.7155	0.4472	1.0000	1.0000
sand	16.35	**0.0026**	**0.0181**	1.0000	1.0000	1.0000	0.0607	**0.0035**	1.0000	1.0000	1.0000	1.0000
Ca [mg/kg]	16.22	**0.0027**	1.0000	**0.0121**	0.0553	1.0000	1.0000	1.0000	1.0000	1.0000	0.1046	0.2055
Mg [mg/kg]	15.46	**0.0038**	0.2619	**0.0412**	**0.0019**	1.0000	1.0000	1.0000	1.0000	1.0000	1.0000	1.0000
P [mg/kg]	8.56	0.073	1.0000	1.0000	0.2200	0.8247	1.0000	0.7326	1.0000	0.3346	1.0000	1.0000
Ece [dS/m]	8.01	0.0913	1.0000	1.0000	1.0000	0.6752	1.0000	1.0000	0.4120	0.5196	1.0000	0.2148
org. mat [%]	7.45	0.1139	1.0000	0.0953	1.0000	1.0000	1.0000	1.0000	1.0000	0.5439	1.0000	1.0000
