# Supplementary material for: Ecology, threats and conservation status of Carex buekii (Cyperaceae) in Central Europe
Source: Sci Rep. 2019 Aug 1;9:11162. doi: 10.1038/s41598-019-47563-0 (PMC6671956; doi:10.1038/s41598-019-47563-0)
Supplement: Supplementary file 4 — Differences in Shannon's index, evenness and number of species in phytocoenoses with Carex bueki [file 41598_2019_47563_MOESM4_ESM.pdf]

Supplementary Table S4

Ecology, threats and conservation status of *Carex buekii* (Cyperaceae) in Central Europe

Helena Więcław[1], Kateřina Šumberová[2], Beata Bosiacka[1], Richard Hrivnák[3], Zygmunt Dajdok[4], Attila Mesterházy[5], Chiara Minuzzo[6], Edoardo Martinetto[7], Jacob Koopman[8]

[1]Institute of Marine & Environmental Sciences, University of Szczecin, Wąska 13, PL-71-415 Szczecin, Poland; [2] The Czech Academy of Sciences, Institute of Botany, Department of Vegetation Ecology, Lidická 25/27, CZ-602 00 Brno, Czech Republic; [3]Institute of Botany, Plant Science and Biodiversity Center, Slovak Academy of Sciences, Dúbravská cesta 9, SK-845 23 Bratislava, Slovakia; [4]Institute of Environmental Biology, University of Wrocław, Kanonia 6/8, PL-50-328 Wrocław, Poland; [5]Directorate of Hortobágy National Park, H-4024 Sumen utca 2, Debrecen, Hungary; [6]Regione Serramonte 10, I-10010 Andrate, Italy, [7]Earth Sciences Department, University of Turin, Via Valperga Caluso 35, I-10125 Turin, Italy, [8]ul. Kochanowskiego 27, PL-73-200 Choszczno, Poland

e-mail: helena.wieclaw@usz.edu.pl

Results of Kruskal–Wallis test and *post hoc* Dunn's multiple comparisons test, showing significance of differences in Shannon's index, evenness and number of species in phytocoenoses with *Carex buekii*. P – Poland, CR - Czech Republic, S – Slovakia, H – Hungary, I – Italy; x̄ – mean; p - statistical significance. Significance level p ≤ 0.05 marked with bold.

Shannon's method	All	Country					Kruskal-Wallis test		Dunn's multiple comparisons test									
		P	CR	S	H	I			P-CR	P-S	P-H	P-I	CR-S	CR-H	CR-I	S-H	S-I	H-I
	x̄	x̄	x̄	x̄	x̄	x̄	H	p	p	p	p	p	p	p	p	p	p	p
Index	2.82	3.20	2.00	2.60	3.42	3.34	31.62	<0.001	**0.001**	0.313	1.000	1.000	0.847	**<0.001**	**0.048**	**0.008**	0.652	1.000
Evenness	0.86	0.92	0.74	0.86	0.90	0.94	30.00	<0.001	**0.001**	**0.005**	1.000	0.736	1.000	0.137	**0.001**	0.335	**0.002**	0.114
Num.Spec.	10	12	6	9	14	11	32.32	<0.001	**0.002**	0.421	1.000	1.000	0.808	**<0.001**	0.134	**0.004**	1.000	1.000
